# Minimalistic bis-triarylpyridinium cations: effective antimicrobials against bacterial and fungal pathogens†

**DOI:** 10.1039/d4md00902a

**Published:** 2025-03-18

**Authors:** Ana M. López-Fernández, Jean C. Neto, Rosa de Llanos, Juan F. Miravet, Francisco Galindo

**Affiliations:** a Departamento de Química Inorgánica y Orgánica, Universitat Jaume I Av. V. Sos Baynat s/n 12071 Castellón Spain francisco.galindo@uji.es; b Unidad Predepartamental de Medicina, Universitat Jaume I Av. V. Sos Baynat s/n 12071 Castellón Spain dellanos@uji.es

## Abstract

A series of twelve compounds from the family of 2,4,6-triarylpyridinium cations have been synthesized, chemically characterized (^1^H, ^13^C NMR, HRMS), and microbiologically evaluated (MIC determination against *S. aureus*, *E. faecalis*, *E. coli*, *P. aeruginosa*, and *C. albicans*). These compounds are quaternary ammonium cations (QACs), classified as either mono-QACs or bis-QACs. The mono-QACs are further divided into those with short (three-carbon) and long (twelve-carbon) pendant chains. An additional structural variable is the number of bromine atoms attached to the aromatic rings, ranging from zero to three. The major findings of this study are: (a) bis-QACs exhibit notably higher antimicrobial activity than mono-QACs; (b) an increased number of bromine atoms on the structure appears to diminish antimicrobial properties and (c) one of the compounds (1a) shows particularly promising properties as a broad spectrum antimicrobial, given its low MICs across all five pathogenic microorganisms studied. Preliminary assays with *C. albicans* show that 1a has a strong mitochondrial activity, causing a remarkable mitochondrial membrane depolarization in this organelle. Taken together, this study positions triarylpyridinium cations—previously unexplored as antimicrobials—as promising candidates for future drug development, especially in light of the growing concern over drug-resistant microorganisms.

## Abstract 2

Triarylpyridinium cations are presented as broad-spectrum antimicrobial agents effective against pathogenic Gram-positive and Gram-negative bacteria, as well as fungi.

## Graphical abstract

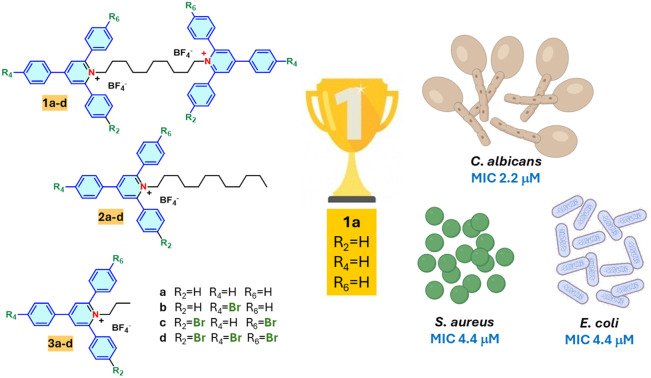

## Introduction

**Fig. 1:**
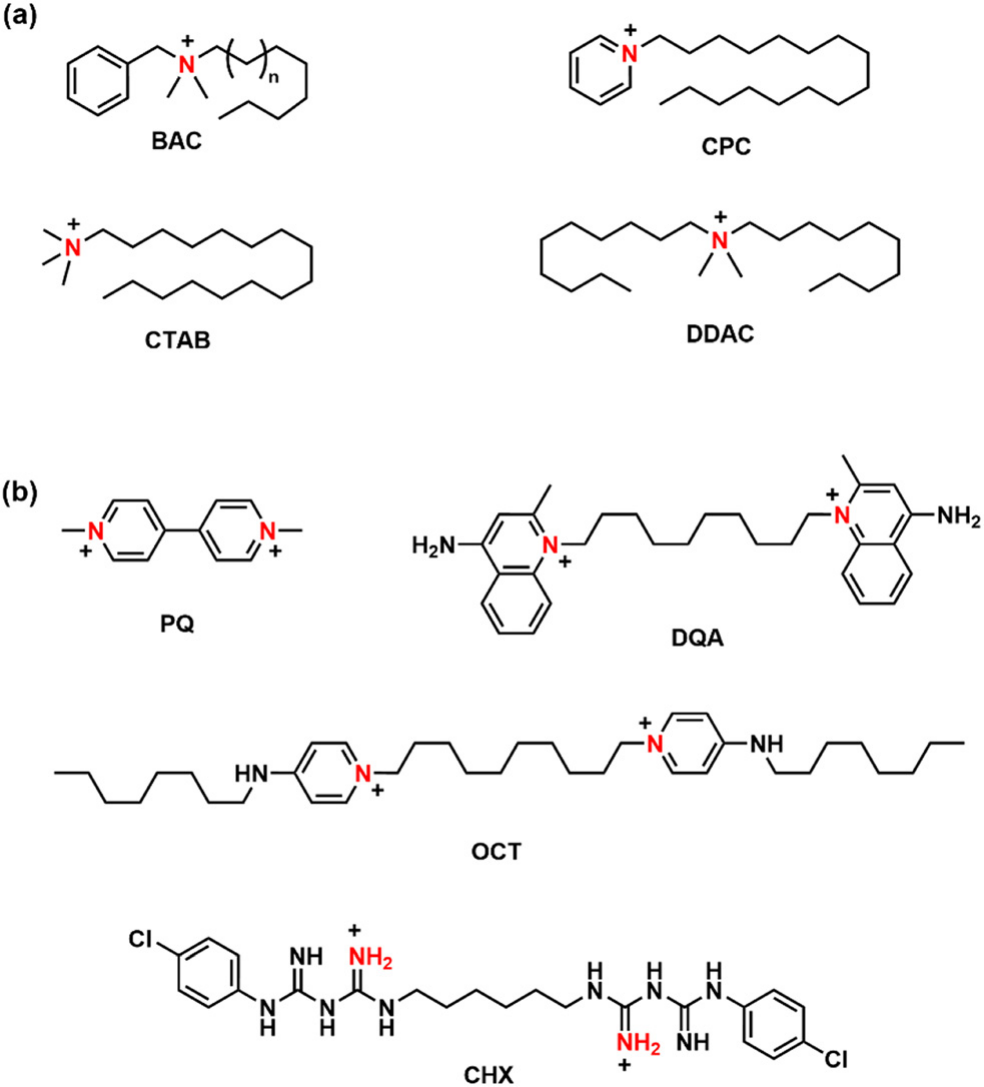
Structures of commercial (a) mono- and (b) bis-QACs.

Organic quaternary ammonium cations (QACs) are well-recognized for their antibacterial and antifungal properties. Since Jacobs identified the bactericidal activity of quaternary salts of hexamethylene in 1916, these compounds have played a critical role in antimicrobial research.^1^ A large variety of molecules and polymers containing nitrogen atoms with a positive charge have been reported.^2–4^ Commercial examples include benzalkonium chloride (BAC), cetyltrimethylammonium chloride (CTAB), cetylpyridinium chloride (CPC), and didecyldimethylammonium chloride (DDAC), all widely used in everyday consumer products such as disinfectants, antiseptic solutions, and surface cleaners. Fig. 1a illustrates the structures of these molecules. The mode of action of these QACs is thought to involve disruption of the microbial membrane through two main interactions: first, the electrostatic attraction between the positively charged QACs and the negatively charged phospholipids, and second, the insertion of the alkyl chain into the lipid bilayer, leading to membrane solubilization or permeabilization. This disruption causes the microbial cell to lose essential intracellular components, including ions, metabolites, and enzymes. Such a lytic mechanism has been proposed for all systems containing phospholipid bilayers, including viruses.^5^ Computer simulations have supported this mechanism as consistent with experimental results.^6^ However, the process may be more complex for microorganisms with difficult-to-penetrate outer membranes, such as Gram-negative bacteria, where an initial permeabilization step is required.^7^ Furthermore, the impact of certain organic cations on fungal cell viability is also associated with the disruption of mitochondrial function.^8–10^

The number of new molecules reported in the literature is steadily increasing, and data collected in recent years have enabled the development of structure–activity relationships (SARs). Initial investigations on monocharged molecules, such as CTAB, BAC, CPC, and DDAC, paved the way for the development of dicharged ammonium derivatives like paraquat (PQ), dequalinium chloride (DQA), octenidine (OCT), and chlorhexidine (CHX), all shown in Fig. 1b. Extensive research on this and related compound classes by Wuest and Minbiole^11–13^ has confirmed that multicharged QACs are generally more potent antimicrobials than their monocharged counterparts.^14^ The search for new compounds featuring non-nitrogen cations, such as phosphonium and sulfonium, is highly active today, given the well-documented microbial resistance to monocationic antiseptics arising from their prolonged use.^15–17^ Additionally, alternative architectures are being investigated as potential options, including pillarenes,^18,19^ ferrocene derivatives,^20^ bis-benzimidazolium cations,^21^ and related systems.^22,23^

A subclass of antimicrobial compounds reported in the literature includes those containing bromine atoms, traditionally studied as metabolites from marine species. For example, the marine bacterium *Pseudoalteromonas phenolica* produces the metabolite bromophene, which exhibits a higher killing rate against methicillin-resistant *Staphylococcus aureus* than vancomycin.^24^ This finding has spurred interest in exploring the antimicrobial activities of brominated derivatives of compounds such as phenols,^25^ resorcinols,^26^ thiophenones,^27^ furanones^28^ and carbazoles.^29^

**Fig. 2:**
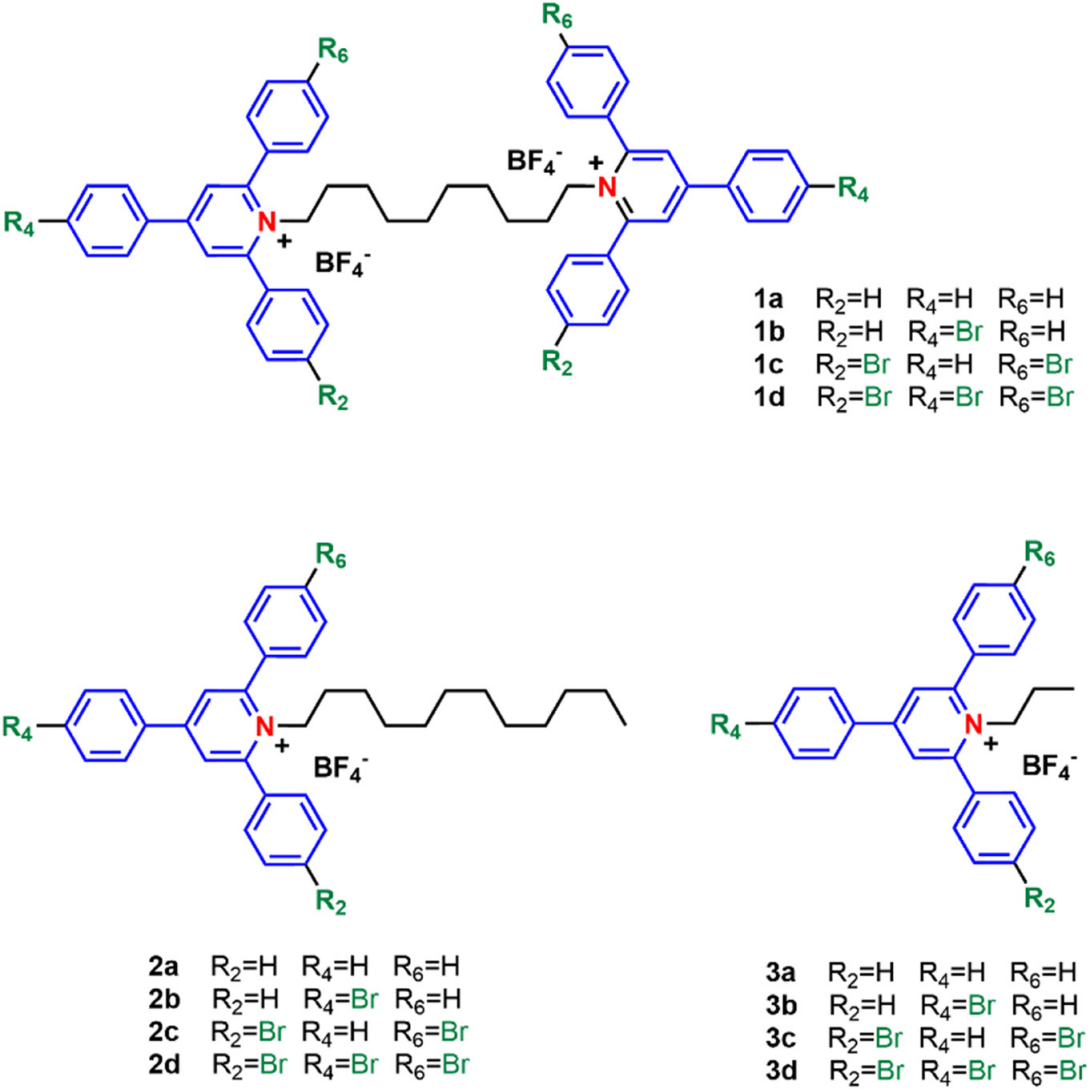
Structure of the 2,4,6-triarylpyridinium derivatives under study.

In this study, we describe the preparation and antimicrobial investigation of a series of 12 quaternary ammonium compounds (QACs) belonging to the pyridinium salt family. All the molecules examined contain a 2,4,6-triarylpyridinium cation, a motif that has not been explored to date in this application area. Notably, this and related scaffolds have been extensively utilized in cell bioimaging,^30–32^ chemical sensing,^33–37^ photocatalysis^38–40^ and the development of optical materials,^41,42^ among other applications. The set of molecules described here (1a–d, 2a–d, 3a–d) is illustrated in Fig. 2. The molecules here presented are either doubly charged (bis-QACs 1a–d, which consist of two cationic units connected by a decamethylene bidge) or singly charged (mono-QACs 2a–d, 3a–d; in this case the length of the alkyl chain attached to the nitrogen atom is also a variable). Additionally, we have studied the effect of bromine atoms at the periphery of the aromatic rings, allowing for classification based on the number of bromine atoms in each cationic motif: no bromine for 1a, 2a, 3a; one bromine for 1b, 2b, 3b; two bromines for 1c, 2c, 3c and three bromines for 1d, 2d, 3d.

We believe that the search for new antimicrobial compounds is more relevant than ever, given the significant threat that drug resistance poses to modern healthcare systems and the economic burden it entails. The resistance of bacteria and fungi to known antimicrobial drugs has been predicted many times to become a significant issue in the future.^43,44^ The list of ESKAPE pathogens, introduced by Rice in 2008,^45^ served as an early warning signal in this context. This concern has been further underscored by the World Health Organization's (WHO) more recent lists of particularly dangerous bacteria (2017, 2024)^46^ and fungi (2022).^47^

## Results and discussion

**Scheme 1:**
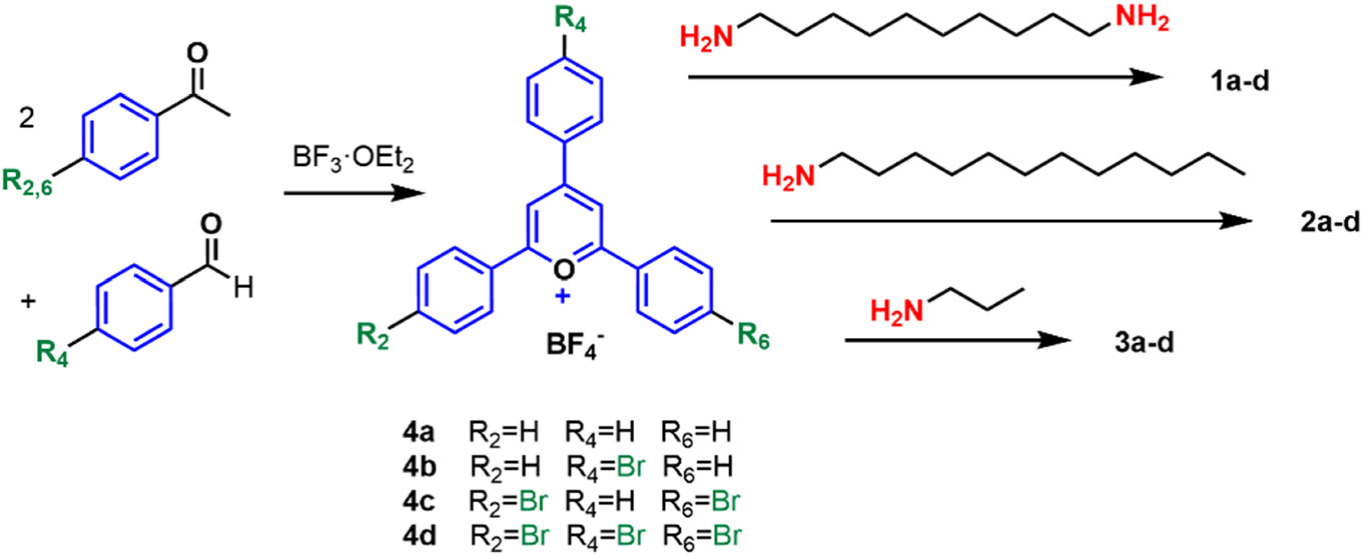
Synthesis of the 2,4,6-triarylpyrylium derivatives 1a–d, 2a–d and 3a–d.

The synthesis of compounds 1a–d, 2a–d, and 3a–d was achieved in just two steps starting from commercial products. The well-known condensation of substituted benzophenones with substituted benzaldehydes produced the corresponding 2,4,6-triarylpyrylium cations 4a–d, as depicted in Scheme 1. These cations were then reacted with 1,10-diaminodecane to yield bis-QACs 1a–d, with 1-aminododecane to produce mono-QACs 2a–d, or with 1-aminopropane to obtain short-chain mono-QACs 3a–d.^48^ All the compounds were characterized using nuclear magnetic resonance spectroscopy (^1^H and ^13^C NMR) and high-resolution mass-spectrometry (HR-MS) (see Fig. S1–S56†).

The antimicrobial activity of compounds 1a–d, 2a–d and 3a–d was tested against five pathogenic species: two Gram-positive bacteria (*Staphylococcus aureus* and *Enterococcus faecalis*), two Gram-negative bacteria (*Escherichia coli* and *Pseudomonas aeruginosa*) and one fungus (*Candida albicans*). Following established literature procedures (see Materials and methods), the minimum inhibitory concentration (MIC) for the 12 compounds was determined. The MIC values for each molecule and microorganism are presented in Table 1. Also standard lysis assays were performed with red blood cells (see also Table 1).

**Table 1:** Minimum inhibitory concentration (MIC_90_) and Lysis_20_ for 1a–d, 2a–d and 3a–d against *S. aureus*, *E. faecalis*, *E. coli*, *P. aeruginosa*, and *C. albicans* and red blood cells, respectively. Concentrations given in μM

Comp.	*S. aureus*	*E. faecalis*	*E. coli*	*P. aeruginosa*	*C. albicans*	Lysis_20_
1a	4.4	1.1	4.4	70	2.2	45
1b	7.6	1.0	7.6	30	118	20
1c	13	13	>103	>103	>103	27
1d	11	6.0	46	>91	>91	>256
2a	28	3.6	28	>112	3.6	31
2b	12	24	25	>200	6.2	26
2c	22	11	178	>178	178	26
2d	40	40	>320	>160	>160	42
3a	146	>293	>585	>293	>293	>256
3b	31	124	>496	>248	>248	>256
3c	54	54	>430	>215	>215	>256
3d	48	24	>380	>190	>90	>256

**Fig. 3:**
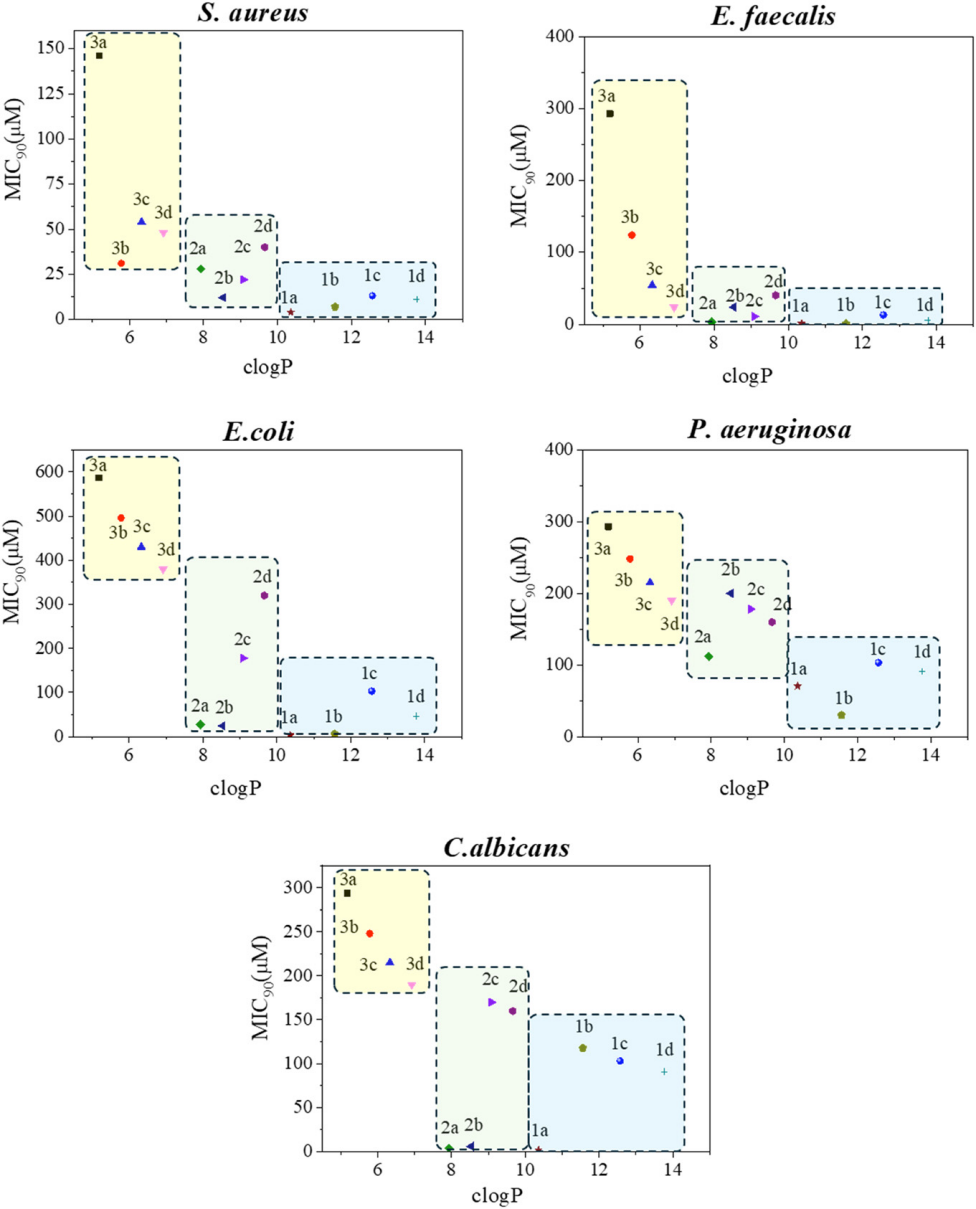
Correlation between MIC and clog *P* of the studied compounds. Relevant points for comparison are those at the bottom of the graph, typically <100 μM, as points at higher concentrations represent the lower detection limits (see details in Table 1).

Several conclusions can be drawn from the data presented in Table 1. Firstly, the efficiency of the doubly charged compounds 1a–d is generally higher than that of the monocharged counterparts. Within the monocharged group, compounds with a longer alkyl chain (dodecyl) exhibit greater activity than those with a shorter chain (propyl). Both observations align with findings reported extensively in the specialized literature.^13^ The greater efficacy of the dodecyl derivatives (2a–d) compared to the propyl cations (3a–d) can be roughly correlated with the hydrophobicity of the compounds.^20^ In our case, the plot of clog *P* (consensus value, calculated according to SwissADME^49^) *versus* MIC is shown in Fig. 3, where it is evident that the compounds in the green boxes (2a–d) are more hydrophobic and effective than those in the yellow areas (3a–d). The same observation applies to the compounds in the blue regions (1a–d); however, in this instance, the electrostatic factor likely plays a significant additional role (double *vs.* single charge). Secondly, among the five microorganisms, Gram-positive bacteria are the most susceptible targets for all types of QACs, which is consistent with the current paradigm.^13^ Thirdly, and most importantly, the number of bromine atoms in the molecules critically affects its antimicrobial efficacy. It has been reported that halogenation can enhance permeation of organic molecules across membranes due to halogen-bonding effects, and numerous drugs use this property. In our investigation into whether the incorporation of bromine atoms into the structure of the tested compounds enhances interactions with microbial cells in a way that amplifies their inhibitory effect, we found that this was not the case. Focusing on the series of bis-QACs, the compound with no bromine (1a) exhibited significantly better activity than the one with three bromine atoms (1d), across the tested microorganisms (MIC 4.4 μM (1a) *vs.* 11 μM (1d) for *S. aureus*; MIC 1.1 μM (1a) *vs.* 6 μM (1d) for *E. faecalis*; 4.4 μM (1a) *vs.* 46 μM (1d) for *E. coli*; 2.2 μM (1a) *vs.* >91 μM (1d) for *C. albicans*). *P. aeruginosa* deserves special mention: of the 12 compounds tested in this study, only dications 1a and 1b exhibited some effect, with moderate MIC values of 70 and 30 μM, respectively. These relatively high concentrations illustrate that this microorganism is notably tenacious, justifying the ongoing research efforts to understand the mechanism of action of bis-cationic antimicrobials. Recent studies have identified efficient molecular bolaamphiphilic architectures capable of targeting the inner membrane of this microorganism.^50^ Future studies on other cationic derivatives could lead to more effective designs, using 1a and 1b as starting points.

The effect of lipophilicity (influenced by the dicationic nature, chain length, and number of bromine atoms) on MIC can be visually observed by examining Fig. 3. As shown, for the series 1a–d (blue box) and 2a–d (green box), the compounds with no bromine or just one bromine (1a, 1b, 2a and 2b) tend to cluster at the bottom of the graph. This is particularly remarkable for *C. albicans* where 1a, 2a and 2b have MIC values as low as 2.2–6.2 μM, indicating significant antifungal activity. Note that Fig. 3 is just for illustrative purposes, as only the values at the bottom of the graph have been estimated with precision. The remaining points indicate the lower limits imposed by the technique (see Table 1 for details).

MIC values found for 1a–d against *S. aureus* fall within the same range as other reported antimicrobials. For instance, DQA, which has a very similar structure to the molecules reported here (aromatic cations separated by a ten-carbon chain), is described to have an MIC of 2.4 μM for *S. aureus*.^2^ For *E. coli*, however, compounds 1a and 1b are more effective than DQA, which is reported to have an MIC of 121 μM.^2^ Similar values have been reported for BAC, another common disinfectant, with MICs of 4 μM for *S. aureus*^12^ and 63 μM for *E. coli*.^12^ In the case of *C. albicans*, the excellent performance of 1a and 2a aligns with the reported values of commercial antifungal agents. For instance, OCT, also a bis-pyridinium compound, is reported to have a MIC of 2.93 μM.^21^ Additionally, for comparative purposes, the MIC for DQA is 5.5 μM against this fungal species.^2^ While methodological differences complicate direct comparisons between reported concentrations, the results presented here demonstrate sufficient internal consistency to affirm that 1a is a promising candidate for future development, as it exhibits high activity against all five tested microorganisms.

This broad-spectrum efficacy does not imply that the operating mechanism is the same for all pathogens. In fact, detailed studies on related bis-pyridinium cations developed by the group of Jolliffe (with some compounds showing MICs as low as 0.7 μM) indicate a detrimental effect of these drugs on the mitochondrial membrane potential of *C. albicans*.^8^ The hypothesis regarding the impact of organic cations on mitochondrial function has gained traction in recent years;^51^ for instance, antifungal cations like xy12 (MIC: 0.24 μM against *C. albicans*^10^) and berberine (MIC: 24 μM against *C. albicans*^52^) also disrupt the functioning of this organelle.

**Fig. 4:**
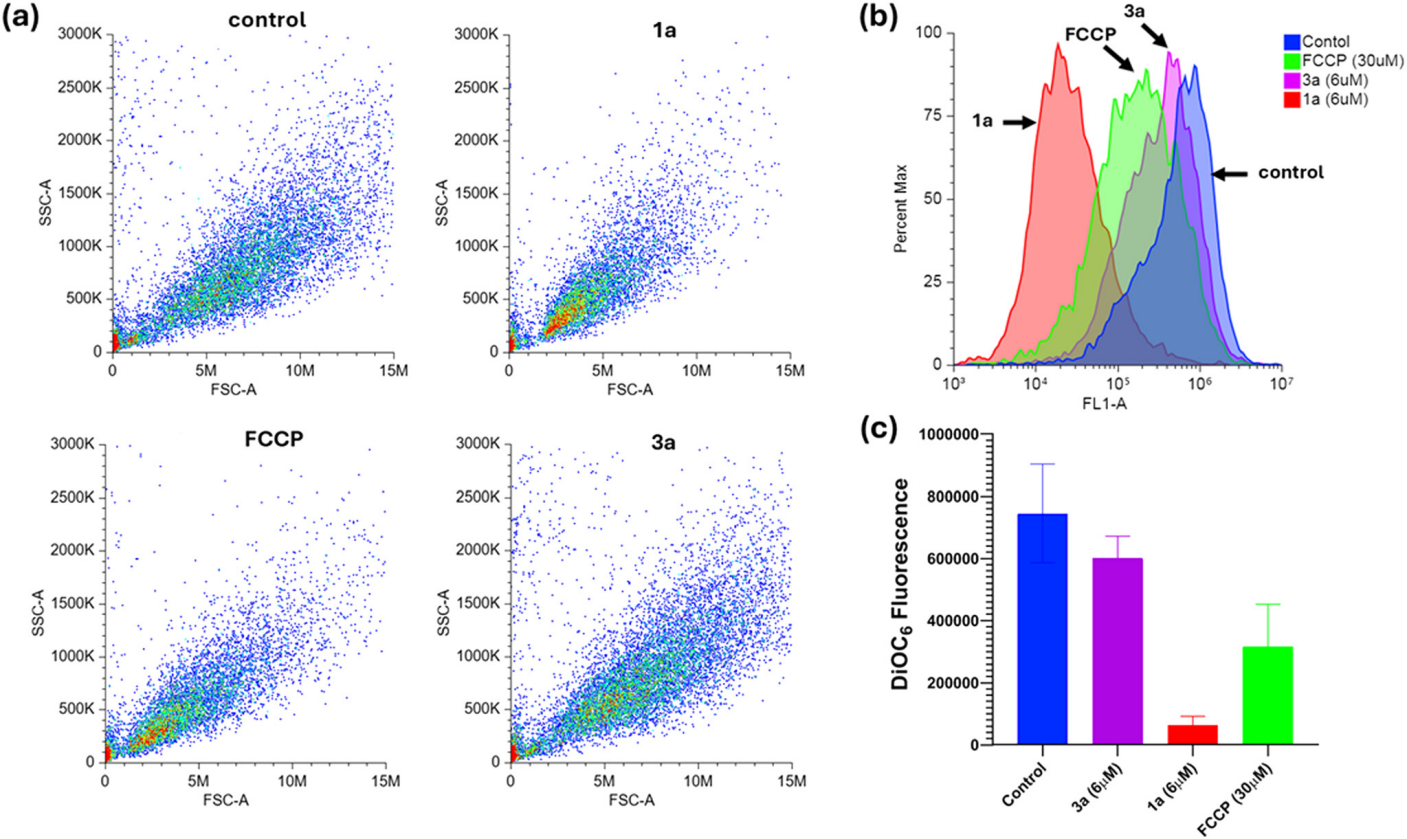
Flow cytometry analysis of cultures of *C. albicans* treated with mitochondrial probe DiOC_6_(3) and several compounds (1a and 3a, and positive control FCCP as a well-known decoupler). (a) Scatter plots. (b) Histogram graph for each sample. (c) Average DiOC_6_(3) fluorescence; values represent the mean ± SD of assays performed in triplicate.

To assess whether our derivatives induce mitochondrial membrane depolarization, an adapted version of an assay described by Obando *et al.*^8^ was conducted. Cultures of *C. albicans* were incubated with the fluorescent probe DiOC_6_(3), which typically accumulates in mitochondria due to the high membrane potential when the organelle works properly. Flow cytometry was used to measure the fluorescence intensity of the accumulated dye, establishing a baseline control (see “control” in Fig. 4). A parallel assay involved *C. albicans* treated previously with the known mitochondrial uncoupler, carbonyl cyanide-4-(trifluoromethoxy)phenylhydrazone (FCCP), at 30 μM. In this case, flow cytometry showed approximately half the fluorescence intensity of the control, indicating mitochondrial membrane depolarization, as expected (Fig. 4). Subsequently, two derivatives, 1a (notable for its antifungal activity) and 3a (inactive), were tested at 6 μM under similar conditions. The fluorescence intensity of DiOC_6_(3) in the case of cells treated with 1a was reduced to about one-tenth of the control, suggesting a potent depolarizing effect on mitochondrial membrane, even more substantial than that of FCCP (used at five times the concentration of 1a). In contrast, 3a-treated cells exhibited normal DiOC_6_(3) accumulation, indicating no impact on mitochondrial membrane potential for this inactive compound.

These assays strongly suggest that certain compounds exhibit activity at this specific mitochondrial site. However, further detailed studies are required to clarify the exact mechanism of inhibition—whether they are targeting a component of the electron transport chain, the ATP synthase complex, or another element.

## Conclusions

In summary, twelve 2,4,6-triarylpyridinium derivatives have been synthesized and characterized. The molecules are classified as bis-quaternary ammonium cations (bis-QACs) or mono-QACs, with varying numbers of bromine atoms (from none to three) on the aromatic core. We investigated the antimicrobial properties of these compounds by determining their MIC values against Gram-positive (*S. aureus* and *E. faecalis*) and Gram-negative (*E. coli* and *P. aeruginosa*) bacteria, as well as a fungus (*C. albicans*). Besides confirming the superior efficacy of bis-QACs over mono-QACs across all microorganisms, we found that, contrary to expectations, the presence of bromine atoms does not enhance antimicrobial action; in some cases, it significantly inhibits it. One compound (1a) shows particularly promising broad-spectrum antimicrobial properties, with low MICs (4.4 μM for *S. aureus*, 1.1 μM for *E. faecalis*, 4.4 μM for *E. coli*, 70 μM for *P. aeruginosa*, and 2.2 μM for *C. albicans*). Preliminary mechanistic studies with *C. albicans* indicate that 1a has a strong activity at the mitochondria, causing a notable depolarization of the membrane of this organelle, opening new avenues for research focusing on targeting this organelle.

Given the threat of drug-resistant microorganisms to healthcare systems and the associated substantial economic burden, the urgent need for new antimicrobials is expected to intensify in the near future. We hope the findings presented here will contribute to this search, as 2,4,6-triarylpyridinium cations have not previously been explored in this context.

## Materials and methods

### Materials

Benzaldehyde, 4-bromoacetophenone, boron trifluoride diethyl etherate and *n*-dodecylamine were purchased from Thermo Scientific. Acetophenone and 4-bromobenzaldehyde were purchased from Sigma-Aldrich. *N*-Propylamine and 1,10-diaminodecane were purchased from Acros Organics. All commercial reagents were used as received.

### Chemical characterization

^1^H and ^13^C NMR spectra were recorded with a 400 MHz Bruker Avance III HD (101 MHz for ^13^C-NMR). High-resolution mass spectra were obtained using a Waters Q-Tof Premier mass spectrometer equipped with an electrospray ionization source. Strains and culture conditions.

The Gram-negative bacterial strain *E. coli* CECT 101 and *P. aeruginosa* ATCC 27853 were supplied by The Colección Española de Cultivos Tipo (CECT, Valencia, Spain) and the American Type Culture Collection (ATCC, Rockville, MD, USA). The Gram-positive bacterial strain *S. aureus* ATCC 25923, *E. faecalis* ATCC 29212 and the fungal strain *C. albicans* ATCC 1023 were supplied by The American Type Culture Collection (ATCC, Maryland, USA). Bacterial and yeast growth was carried out aerobically overnight at 35 °C in Mueller Hinton agar and Sabouraud dextrose agar, respectively (Scharlau, Spain).

### Minimum inhibitory concentration (MIC)

The MIC of the compounds 1a–d, 2a–d, and 3a–d was determined by a broth microdilution method according to the EUCAST protocol. Briefly, 0.5 McFarland bacterial and fungal suspensions were prepared in Müeller Hinton broth (MHB) and Sabouraud dextrose broth (SDB) media, respectively. In a 96-well plate, serial dilutions of compounds 1a–d, 2a–d, 3a–d starting from 512 to 0 μg mL^−1^ were made in MHB and SDB media, respectively. After incubation overnight at 37 °C, results were evaluated according to turbidity. Bacterial growth was determined by measuring absorbance at a wavelength of 600 nm and at 520 nm for fungi.^53–55^

### Lysis assays

The hemolysis test was performed following literature procedures.^14^ Preparation of the erythrocyte suspension: human blood (6 mL) was transferred to a Falcon tube. The sample was then centrifuged at 10 000 rpm for 10 minutes. After centrifugation, the supernatant was discarded, and the cells were resuspended in 4 mL of 10 mM PBS. This centrifugation and resuspension process was repeated twice to remove plasma residues. Finally, the resulting cell suspension was diluted at a 1 : 20 ratio with 10 mM PBS. Hemolysis assay: a total of 500 μL of the prepared erythrocyte suspension was aliquoted into Eppendorf tubes according to the experimental conditions. The final DMSO concentration in each condition was adjusted to 1%. A 1% Triton™ X-100 solution was used as the positive control (100% hemolysis), while the negative control consisted of erythrocytes suspended in PBS with 1% DMSO (0% hemolysis). Samples were incubated at 37 °C with shaking at 200 rpm for 1 hour. Following incubation, the samples were centrifuged at 10 000 rpm for 10 minutes. Subsequently, 200 μL of the supernatant was collected and transferred to a 96-well plate. Absorbance was measured at 520 nm using a plate reader.

### Evaluation of the mitochondrial function of *C. albicans* using DiOC_6_ staining

The methodology was conducted as described by Obando *et al.*, with some modifications.^8^ To evaluate mitochondrial function in *C. albicans* by DiOC_6_ staining, the yeast cells were initially cultivated overnight in Sabouraud broth. The cultures were then transferred to a fresh medium, adjusting the OD_600_ to 0.1. Experimental treatments included exposure to compounds 1a and 3a at a final concentration of 6 μM, as well as the positive control FCCP at 30 μM. The cultures were incubated for 3.5 hours with orbital shaking at 37 °C. After this period, the OD of each culture was standardized to 0.06 using the corresponding spent medium. Cells were subsequently stained with 0.3 μM DiOC_6_ for 20 minutes. Assays were performed in triplicate. Analysis was conducted using a BD Accuri™ C6 flow cytometer.

### Synthesis of precursor pyrylium salts (4a–d)

The synthesis of pyridinium salts was carried out using the previously described procedure with some modifications.^30^ The general method involves the addition of the corresponding acetophenone (2 equiv.) and the *p*-substituted benzaldehyde (1 equiv.) along with boron trifluoride etherate (BF_3_·OEt_2_) (2.4 equiv.). The reactions were performed under reflux in toluene with stirring under an inert atmosphere for 24 hours. Continuedly, the reactive mixture was cooled to room temperature and precipitated with diethyl ether. The obtained precipitate was vacuum filtered, washed three times with diethyl ether, and dried under vacuum.

#### 4a

Starting reagents: benzaldehyde (2.4 mL, 23.32 mmol), acetophenone (5.6 mL, 41.18 mmol). After precipitation: yellow solid, yield: 2.590 g, 28%. ^1^H NMR (400 MHz, DMSO-d_6_) *δ* (ppm) 9.17 (s, 2H), 8.60 (d, *J* = 7.2 Hz, 6H), 7.88 (t, *J* = 7.3 Hz, 3H), 7.83–7.75 (m, 6H); ^13^C NMR (101 MHz, DMSO-d_6_) *δ* (ppm) 170.09, 165.13, 135.15, 135.00, 132.49, 130.01, 129.85, 129.81, 129.12, 128.80, 115.20.

#### 4b

Starting reagents: bromobenzaldehyde (2 g, 10.59 mmol), acetophenone (2.5 mL, 21.43 mmol). After precipitation: yellow solid, yield: 1.547 g, 31%. ^1^H NMR (400 MHz, DMSO-d_6_) *δ* (ppm) 9.17 (s, 2H), 8.57 (dd, *J* = 9.0, 7.4 Hz, 6H), 8.03 (d, *J* = 8.8 Hz, 2H), 7.88 (t, *J* = 7.3 Hz, 2H), 7.80 (t, *J* = 7.6 Hz, 4H). ^13^C NMR (101 MHz, DMSO-d_6_) *δ* (ppm) 170.23, 163.88, 135.10, 132.88, 131.78, 131.61, 129.86, 129.09, 128.83, 115.16.

#### 4c

Starting reagents: benzaldehyde (486 μL, 4.7 mmol), 4-bromoacetophenone (2.01 g, 9,9 mmol). After precipitation: yellow solid, yield: 826 mg, 32%. ^1^H NMR (400 MHz, DMSO-d_6_) *δ* (ppm) 9.20 (s, 2H), 8.60 (d, *J* = 7.3 Hz, 2H), 8.52 (d, *J* = 8.8 Hz, 6H), 8.02 (d, *J* = 8.7 Hz, 4H), 7.88 (t, *J* = 7.4 Hz, 1H), 7.79 (t, *J* = 7.6 Hz, 2H). ^13^C NMR (101 MHz, DMSO-d_6_) *δ* (ppm) 169.27, 165.27, 135.40, 132.91, 132.34, 131.74, 130.57, 130.12, 130.07, 129.85, 129.54, 128.27, 115.59.

#### 4d

Starting reagents: 4-bromobenzaldehyde (2 g, 10.59 mmol), 4-bromoacetophenone (4.31 g, 21.428 mmol). After precipitation: yellow solid, yield: 1.146 g, 17%. ^1^H NMR (400 MHz, DMSO-d_6_) *δ* (ppm) 9.20 (s, 2H), 8.53 (dd, *J* = 8.9, 6.8 Hz, 6H), 8.03 (dd, *J* = 5.3, 3.4 Hz, 6H). ^13^C NMR (101 MHz, DMSO-d_6_) *δ* (ppm) 169.41, 164.02, 132.93, 131.85, 131.75, 131.50, 131.45, 130.60, 129.66, 128.24, 115.54.

### Synthesis of bis-pyridinium salts (1a–d)

The previously prepared pyrylium salts 4a–d (2.2 equiv.) are dissolved with triethylamine (2 equiv.) and 1,10-diaminodecane (1 equiv.) in absolute ethanol. The solution is refluxed overnight under a nitrogen atmosphere. The solvent is removed under vacuum and precipitated with diethyl ether. The precipitate is then redissolved in a minimum amount of chloroform and reprecipitated with diethyl ether. Finally, the product is subjected to ultrasonication to obtain the desired product.

#### 1a

Starting reagents: 4a (432 mg, 1.09 mmol), 1,10-diaminodecane (88 mg, 0.49 mmol). After precipitation: white solid, yield: 459 mg, 99%. ^1^H NMR (400 MHz, CDCl_3_) *δ* (ppm) 7.87 (s, 4H), 7.83–7.74 (m, 12H), 7.64–7.52 (m, 12H), 7.56–7.47 (m, 6H), 4.39 (t, *J* = 8.2 Hz, 4H), 1.42 (q, *J* = 7.8 Hz, 4H), 0.74–0.57 (m, 12H). ^13^C NMR (101 MHz, CDCl_3_) *δ* (ppm) 156.68, 155.92, 134.21, 132.93, 132.22, 131.14, 129.87, 129.45, 129.24, 128.28, 126.91, 55.01, 29.58, 28.22, 27.42, 25.97. HRMS (ESI-TOF)^+^ calcd. for C_56_H_54_N_2_^2+^ (M^2+^) (*m*/*z*): 377.2144. Found (M^2+^) (*m*/*z*): 377.2137.

#### 1b

Starting reagents: 4b (588 mg, 1.24 mmol), 1,10-diaminodecane (100 mg, 0.56 mmol). After precipitation: yellow solid, yield: 460 mg, 75%. ^1^H NMR (400 MHz, CDCl_3_) *δ* (ppm) 7.83 (s, 4H), 7.80–7.76 (m, 8H), 7.65 (s, 8H), 7.57 (m, 12H), 4.35 (t, *J* = 8.1 Hz, 4H), 1.49–1.39 (m, 4H), 0.72–0.56 (m, 12H); ^13^C NMR (101 MHz, CDCl_3_) *δ* (ppm) 156.79, 154.78, 133.23, 133.12, 132.83, 131.20, 129.76, 129.45, 129.20, 127.29, 126.85, 55.10, 29.48, 28.15, 27.40, 25.93; HRMS (ESI-TOF)^+^ calcd. for C_56_H_52_Br_2_N_2_^2+^ (M^2+^) (*m*/*z*): 455.1249. Found (M^2+^) (*m*/*z*): 455.1245.

#### 1c

Starting reagents: 4c (605 mg, 1.09 mmol), 1,10-diaminodecane (88 mg, 0.49 mmol). After precipitation: brown precipitate, yield: 609 mg, 96%. ^1^H NMR (400 MHz, CDCl_3_) *δ* (ppm) 7.81 (s, 4H), 7.75–7.67 (m, 20H), 7.57–7.47 (m, 6H), 4.32 (t, *J* = 8.3 Hz, 4H), 1.42 (p, *J* = 7.5 Hz, 4H), 0.83–0.59 (m, 12H); ^13^C NMR (101 MHz, CDCl_3_) *δ* (ppm) 156.15, 155.68, 133.97, 132.70, 132.42, 131.67, 131.02, 129.91, 128.28, 127.05, 126.04, 55.14, 29.40, 28.19, 27.35, 25.92; HRMS (ESI-TOF)^+^ calcd. for C_56_H_50_Br_4_N_2_^2+^ (M^2+^) (*m*/*z*): 533.0354. Found (M^2+^) (*m*/*z*): 533.0347.

#### 1d

Starting reagents: 4d (533 mg, 0.84 mmol), 1,10-diaminodecane (68 mg, 0.4 mmol). After precipitation: brown precipitate, yield: 441 mg, 80%. ^1^H NMR (400 MHz, DMSO-d_6_) *δ* (ppm) 8.51 (s, 4H), 8.19 (d, *J* = 8.4 Hz, 4H), 7.92 (d, *J* = 8.2 Hz, 8H), 7.83 (d, *J* = 8.0 Hz, 4H), 7.78 (d, *J* = 8.2 Hz, 8H), 4.25 (t, *J* = 8.3 Hz, 4H), 1.38–1.31 (m, 4H), 0.79–0.53 (m, 12H); ^13^C NMR (101 MHz, DMSO-d_6_) *δ* (ppm) 154.90, 152.91, 132.56, 132.17, 132.03, 131.89, 131.32, 130.58, 126.69, 126.31, 124.77, 54.20, 45.78, 28.33, 28.01, 27.11, 25.14, 8.65; HRMS (ESI-TOF)^+^ calcd. for C_56_H_48_Br_6_N_2_^2+^ (M^2+^) (*m*/*z*): 610.9459. Found (M^2+^) (*m*/*z*): 610.9455.

### Synthesis of pyridinium salts (2a–d, 3a–d)

The corresponding amine (1.3 equiv.) with the previous pyrylium salts 4a–d (1 equiv.) is dissolved in absolute ethanol. The solution is refluxed overnight under a nitrogen atmosphere. The solvent is removed under vacuum and precipitated with diethyl ether. The precipitate is then redissolved in a minimum amount of chloroform and reprecipitated with diethyl ether or hexane. Finally, the product is subjected to ultrasonication to obtain the desired product.

#### 2a

Starting reagents: 4a (300 mg, 0.76 mmol), *n*-dodecylamine (188 mg, 0.98 mmol). After precipitation: yellowish oil, yield: 379 mg, 89%. ^1^H NMR (400 MHz, CDCl_3_) *δ* (ppm) 7.85 (s, 2H), 7.82–7.77 (m, 4H), 7.75 (d, *J* = 7.7 Hz, 2H), 7.60 (t, *J* = 5.6 Hz, 6H), 7.57–7.47 (m, 3H), 4.40 (t, *J* = 8.3 Hz, 2H), 1.52–1.36 (m, 2H), 1.34–1.06 (m, 10H), 1.01 (q, *J* = 6.4 Hz, 2H), 0.97–0.84 (m, 5H), 0.75 (q, *J* = 3.5 Hz, 4H); ^13^C NMR (101 MHz, CDCl_3_) *δ* (ppm) 156.65, 155.87, 134.23, 132.97, 132.17, 131.13, 129.83, 129.42, 129.21, 128.25, 126.86, 54.89, 32.02, 29.79, 29.68, 29.58, 29.43, 29.36, 29.00, 28.07, 26.11, 22.80, 14.23; HRMS (ESI-TOF)^+^ calcd. for C_35_H_42_N^+^ (M^+^) (*m*/*z*): 476.3317. Found (M^+^) (*m*/*z*): 476.3314.

#### 2b

Starting reagents: 4b (300 mg, 0.63 mmol), *n*-dodecylamine (157 mg, 0.82 mmol). After precipitation: brown oil, yield: 290 mg, 72%. ^1^H NMR (400 MHz, CDCl_3_) *δ* (ppm) 7.83 (s, 2H), 7.81–7.74 (m, 4H), 7.63 (s, 4H), 7.61–7.56 (m, 6H), 4.37 (t, *J* = 8.5 Hz, 2H), 1.50–1.37 (m, 2H), 1.34–1.06 (m, 10H), 1.00 (q, *J* = 6.9 Hz, 2H), 0.94–0.82 (m, 5H), 0.73 (p, *J* = 3.5 Hz, 4H). ^13^C NMR (101 MHz, CDCl_3_) *δ* (ppm) 156.78, 154.74, 133.25, 133.08, 132.84, 131.19, 129.75, 129.43, 129.17, 127.22, 126.79, 54.99, 32.02, 29.75, 29.68, 29.57, 29.43, 29.36, 28.99, 28.05, 26.13, 22.80, 14.24; HRMS (ESI-TOF)^+^ calcd. for C_35_H_41_BrN^+^ (M^+^) (*m*/*z*): 554.2422. Found (M^+^) (*m*/*z*): 554.2417.

#### 2c

Starting reagents: 4c (300 mg, 0.54 mmol), *n*-dodecylamine (135 mg, 0.70 mmol). After precipitation: brown oil, yield: 272 mg, 70%. ^1^H NMR (400 MHz, CDCl_3_) *δ* (ppm) 7.83 (s, 2H), 7.76–7.70 (m, 6H), 7.71–7.66 (m, 4H), 7.60–7.48 (m, 3H), 4.35 (t, *J* = 8.0 Hz, 2H), 1.42 (p, *J* = 6.2 Hz, 2H), 1.33–1.02 (m, 11H), 0.99–0.74 (m, 10H); ^13^C NMR (101 MHz, CDCl_3_) *δ* (ppm) 156.21, 155.72, 133.93, 132.77, 132.46, 131.60, 130.87, 129.93, 128.26, 127.05, 126.16, 55.00, 32.05, 29.80, 29.73, 29.66, 29.47, 29.44, 29.08, 28.16, 26.13, 22.82, 14.25; HRMS (ESI-TOF)^+^ calcd. for C_35_H_40_Br_2_N^+^ (M^+^) (*m*/*z*): 632.1528. Found (M^+^) (*m*/*z*): 632.1533.

#### 2d

Starting reagents: 4d (300 mg, 0.47 mmol), *n*-dodecylamine (118 mg, 0.62 mmol). After precipitation: brown oil, yield: 185 mg, 49%. ^1^H NMR (400 MHz, DMSO-d_6_) *δ* (ppm) 8.51 (s, 2H), 8.19 (d, *J* = 8.4 Hz, 2H), 7.93 (d, *J* = 8.0 Hz, 4H), 7.84 (d, *J* = 8.2 Hz, 4H), 7.79 (d, *J* = 8.6 Hz, 2H), 4.27 (t, *J* = 6.1 Hz, 2H), 1.36 (p, *J* = 6.7 Hz, 2H), 1.29–1.08 (m, 12H), 0.95–0.68 (m, 9H); ^13^C NMR (101 MHz, DMSO-d_6_) *δ* (ppm) 154.95, 152.88, 132.54, 132.18, 132.05, 131.90, 131.33, 130.59, 126.66, 126.30, 124.78, 54.30, 31.24, 28.96, 28.83, 28.72, 28.62, 28.36, 28.15, 27.24, 25.03, 22.04, 13.91; HRMS (ESI-TOF)^+^ calcd. for C_35_H_39_Br_3_N^+^ (M^+^) (*m*/*z*): 710.0633. Found (M^+^) (*m*/*z*): 710.0627.

#### 3a

Starting reagents: 4a (300 mg, 0.76 mmol), *n*-propylamine (81 μL, 0.99 mmol). After precipitation: white precipitate, yield: 307 mg, 93%. ^1^H NMR (400 MHz, CDCl_3_) *δ* (ppm) 7.85 (s, 2H), 7.82–7.77 (m, 4H), 7.75 (d, *J* = 6.8 Hz, 2H), 7.60 (t, *J* = 2.9 Hz, 6H), 7.57–7.46 (m, 3H), 4.37 (t, *J* = 8.2 Hz, 2H), 1.47 (m, 2H), 0.41 (t, *J* = 7.4 Hz, 3H); ^13^C NMR (101 MHz, CDCl_3_) *δ* (ppm) 156.71, 155.91, 134.22, 132.98, 132.18, 131.15, 129.83, 129.44, 129.17, 128.26, 126.87, 56.37, 23.60, 10.86; HRMS (ESI-TOF)^+^ calcd. for C_26_H_24_N^+^ (M^+^) (*m*/*z*): 350.1909. Found (M^+^) (*m*/*z*): 350.1904.

#### 3b

Starting reagents: 4b (300 mg, 0.63 mmol), *n*-propylamine (68 μL, 0.82 mmol). After precipitation: yellow precipitate, yield: 292 mg, 90%. ^1^H NMR (400 MHz, CDCl_3_) *δ* (ppm) 7.84 (s, 2H), 7.82–7.72 (m, 4H), 7.64 (s, 4H), 7.60 (dd, *J* = 5.0, 1.9 Hz, 6H), 4.34 (t, *J* = 8.6 Hz, 2H), 1.56–1.41 (m, 2H), 0.41 (t, *J* = 7.4 Hz, 3H); ^13^C NMR (101 MHz, CDCl_3_) *δ* (ppm) 156.85, 154.82, 133.23, 133.10, 131.24, 129.76, 129.47, 129.11, 127.27, 126.82, 56.46, 23.60, 10.87; HRMS (ESI-TOF)^+^ calcd. for C_26_H_23_BrN^+^ (M^+^) (*m*/*z*): 4281014. Found (M^+^) (*m*/*z*): 428.1013.

#### 3c

Starting reagents: 4c (300 mg, 0.54 mmol), *n*-propylamine (58 μL, 0.71 mmol). After precipitation: white precipitate, yield: 108 mg, 36%. ^1^H NMR (400 MHz, CDCl_3_) *δ* (ppm) 7.82 (s, 2H), 7.76–7.64 (m, 10H), 7.61–7.45 (m, 3H), 4.31 (t, *J* = 7.0 Hz, 2H), 1.53–1.39 (m, 2H), 0.47 (t, *J* = 7.4 Hz, 3H); ^13^C NMR (101 MHz, CDCl_3_) *δ* (ppm) 156.22, 155.76, 133.93, 132.78, 132.45, 131.65, 130.84, 129.92, 128.27, 127.06, 126.14, 56.50, 23.57, 10.94; HRMS (ESI-TOF)^+^ calcd. for C_26_H_22_Br_2_N^+^ (M^+^) (*m*/*z*): 506.0119. Found (M^+^) (*m*/*z*): 506.0116.

#### 3d

Starting reagents: 4d (300 mg, 0.47 mmol), *n*-propylamine (51 μL, 0.62 mmol). After precipitation: brown precipitate, yield: 158 mg, 50%. ^1^H NMR (400 MHz, CDCl_3_) *δ* (ppm) 7.81 (s, 2H), 7.72 (d, *J* = 8.2 Hz, 4H), 7.66 (d, *J* = 8.3 Hz, 4H), 7.62 (d, *J* = 7.2 Hz, 4H), 4.29 (t, *J* = 8.1 Hz, 2H), 1.46 (p, *J* = 7.5 Hz, 2H), 0.47 (t, *J* = 7.3 Hz, 3H); ^13^C NMR (101 MHz, CDCl_3_) *δ* (ppm) 155.90, 155.10, 133.19, 132.90, 132.80, 131.50, 130.79, 129.74, 127.60, 126.98, 126.24, 56.58, 23.54, 10.95; HRMS (ESI-TOF)^+^ calcd. for C_26_H_21_Br_3_N^+^ (M^+^) (*m*/*z*): 583.9224. Found (M^+^) (*m*/*z*): 583.9222.

## Author contributions

AMLF: investigation, methodology; JCN: investigation, methodology; RdL: funding acquisition, supervision, writing – review & editing; JFM: funding acquisition, supervision; FG: conceptualization, funding acquisition, supervision, writing – original draft, writing – review & editing.

## Conflicts of interest

There are no conflicts to declare.

## Supplementary Material

MD-016-D4MD00902A-s001

## Data Availability

The data supporting this article have been included as part of the ESI.†
