# Supplementary material for: Minimalistic bis-triarylpyridinium cations: effective antimicrobials against bacterial and fungal pathogens
Source: RSC Med Chem. 2025 Mar 18;16(6):2641–50. doi: 10.1039/d4md00902a (PMC11969996; doi:10.1039/d4md00902a)
Supplement: MD-016-D4MD00902A-s001 [file MD-016-D4MD00902A-s001.pdf]

Supplementary Information (SI) for RSC Medicinal Chemistry.
This journal is © The Royal Society of Chemistry 2025

Minimalistic bis-triarylpyridinium cations: effective antimicrobials against bacterial and fungal pathogens

Ana M. López-Fernández,[a] Jean C. Neto [a], Rosa de Llanos,*[b] Juan F. Miravet,[a] Francisco Galindo*[a]

(a) Departamento de Química Inorgánica y Orgánica, Universitat Jaume I, Av. V. Sos Baynat s/n, 12071, Castellón, Spain. E-mail: francisco.galindo@uji.es

(b) Unidad Predepartamental de Medicina, Universitat Jaume I, Av. V. Sos Baynat s/n, 12071, Castellón, Spain. E-mail: dellanos@uji.es

Table of Contents

Spectroscopic data of compounds 2

Determination of MICs for all the studied compounds 30

Spectroscopic data of compounds

2,4,6-triphenylpyrylium tetrafluoroborate (4a)

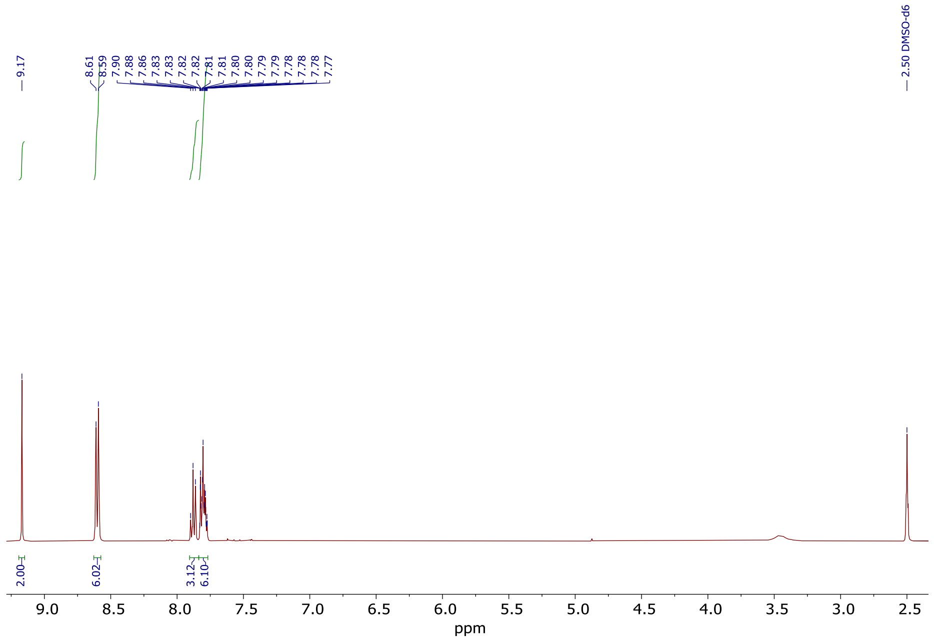

Figure S1. ^{1}H NMR (DMSO-d_6) spectra of the precursor *2,4,6-triphenylpyrylium tetrafluoroborate (**4a**).*

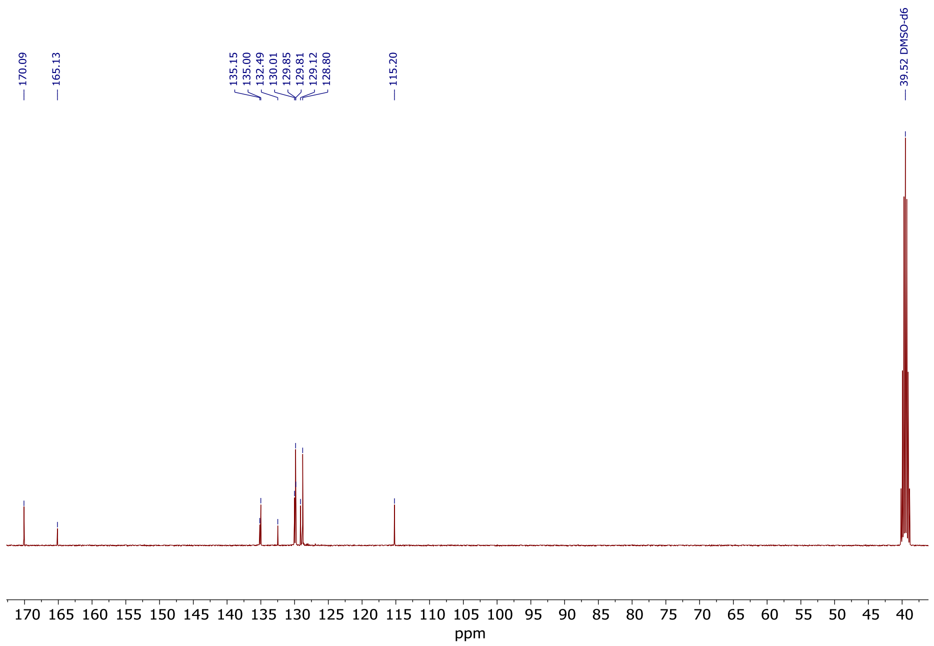

Figure S2. ^{13}C NMR (DMSO-d_6) spectra of the precursor *2,4,6-triphenylpyrylium tetrafluoroborate (**4a**).*

4-(4-bromophenyl)-2,6-diphenylpyrylium tetrafluoroborate (4b)

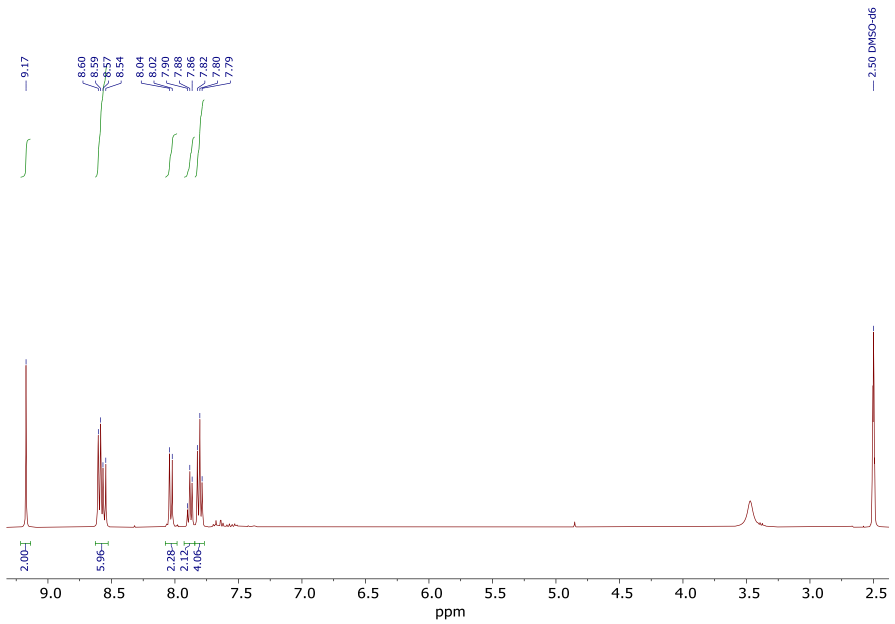

Figure S3. ^{1}H NMR (DMSO-d_6) spectra of the precursor *4-(4-bromophenyl)-2,6-diphenylpyrylium tetrafluoroborate (**4b**).*

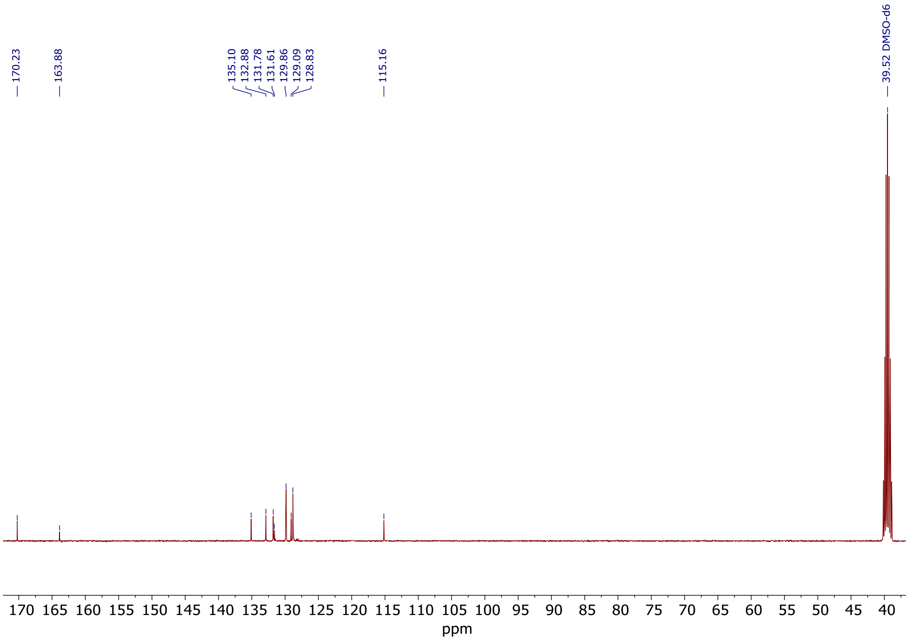

Figure S4. ^{13}C NMR (DMSO-d_6) spectra of the precursor *4-(4-bromophenyl)-2,6-diphenylpyrylium tetrafluoroborate (**4b**).*

2,6-bis(4-bromophenyl)-4-phenylpyrylium tetrafluoroborate (4c)

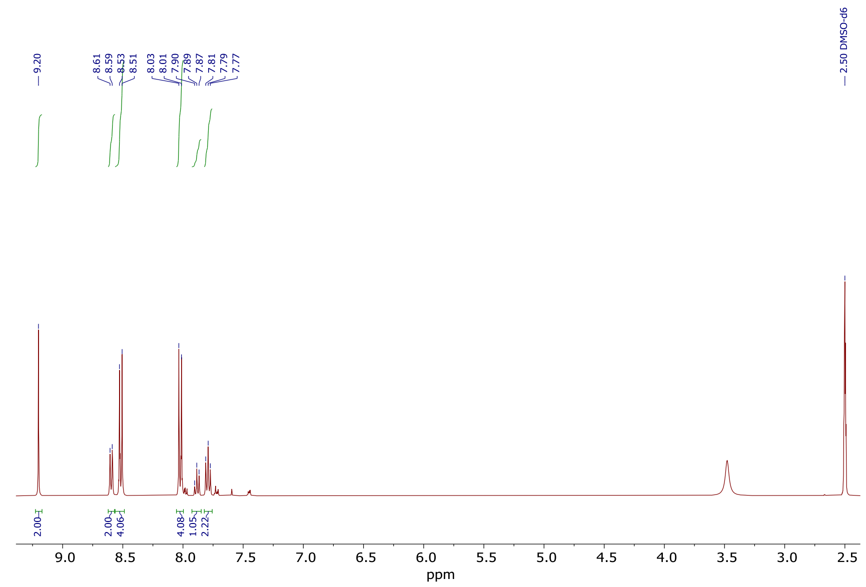

Figure S5. ^{1}H NMR (DMSO-d_6) spectra of the precursor *2,6-bis(4-bromophenyl)-4-phenylpyrylium tetrafluoroborate (**4c**).*

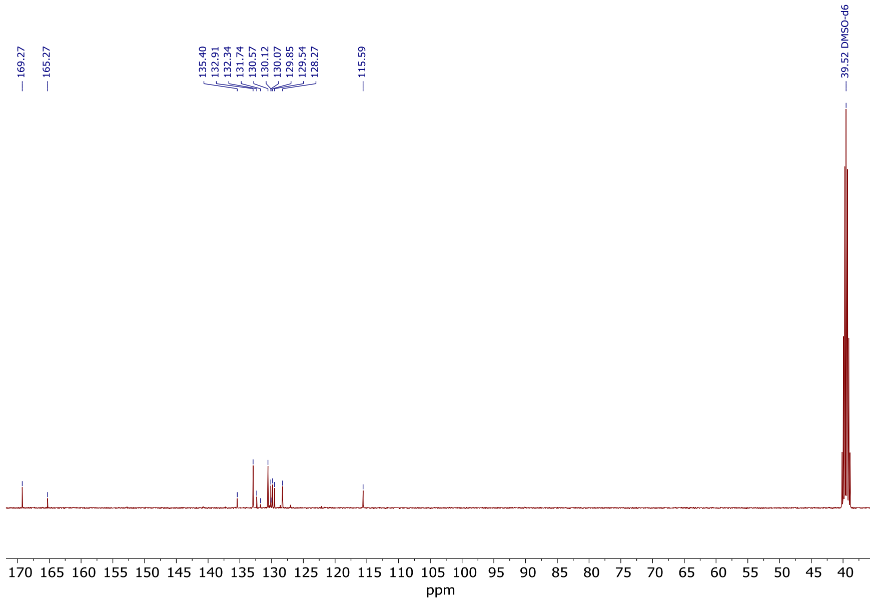

Figure S6. ^{13}C NMR (DMSO-d_6) spectra of the precursor *2,6-bis(4-bromophenyl)-4-phenylpyrylium tetrafluoroborate (**4c**).*

2,4,6-tris(4-bromophenyl)pyrylium tetrafluoroborate (4d)

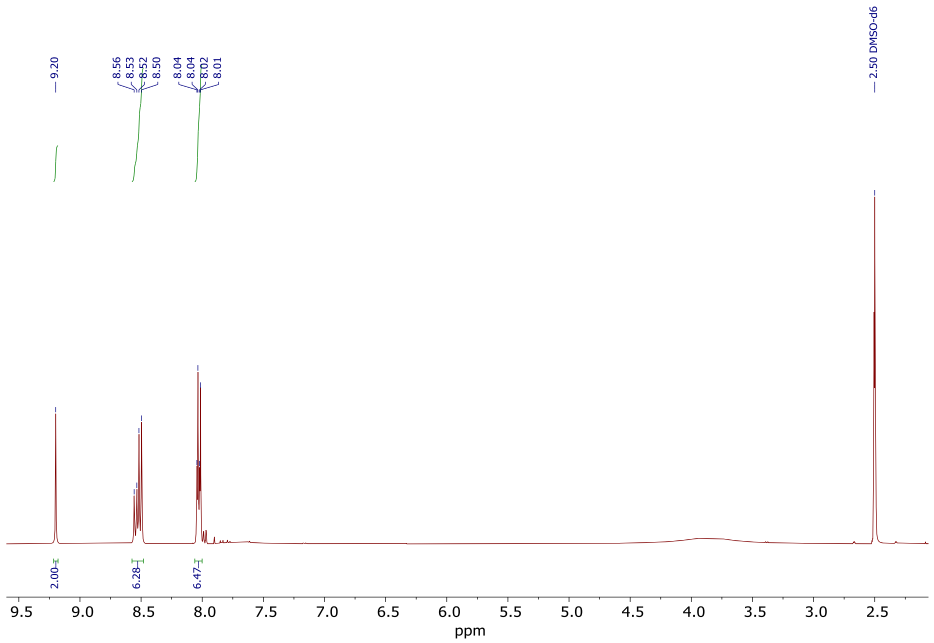

Figure S7. ^{1}H NMR (DMSO-d$_6$) spectra of the precursor *2,4,6-tris(4-bromophenyl)pyrylium tetrafluoroborate (**4d**).*

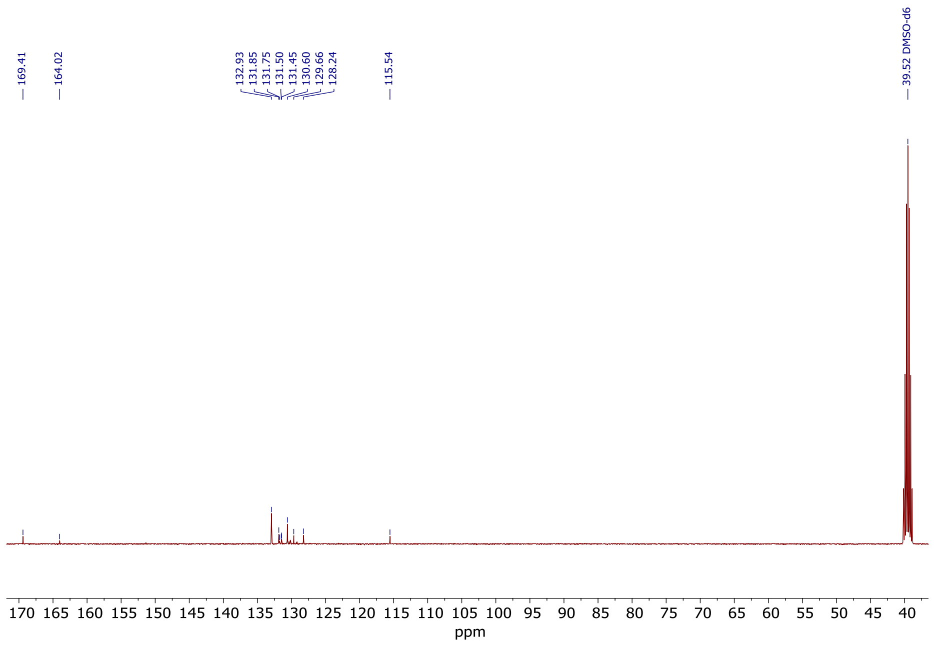

Figure S8. ^{13}C NMR (DMSO-d$_6$) spectra of the precursor *2,4,6-tris(4-bromophenyl)pyrylium tetrafluoroborate (**4d**).*

Compound 1a

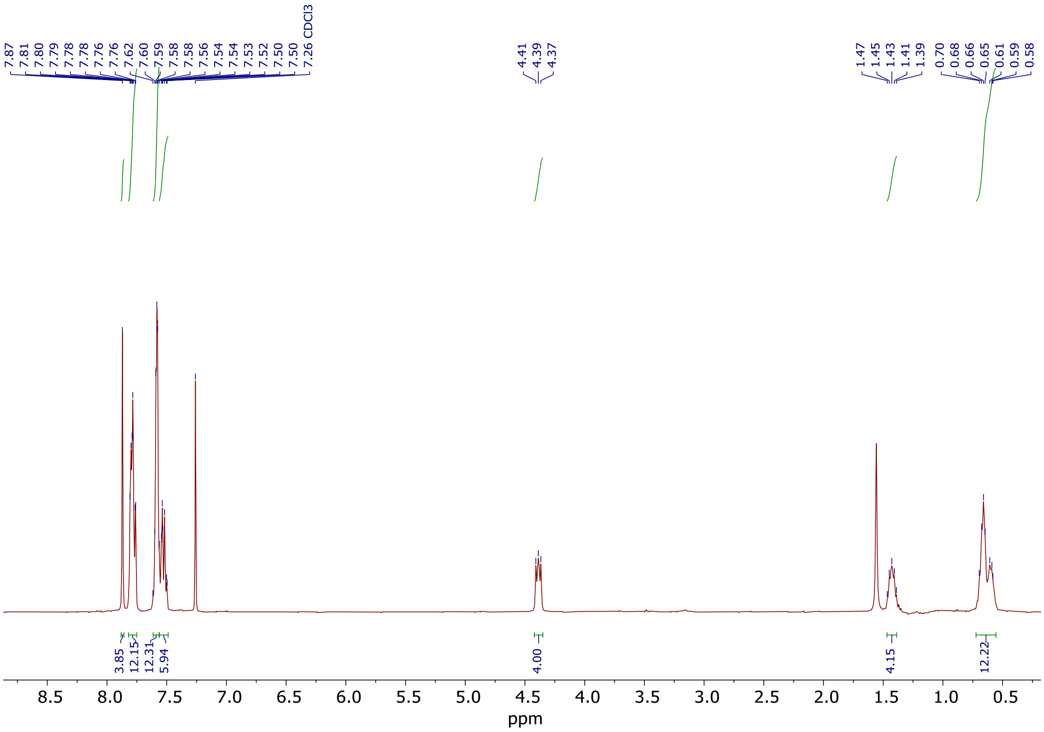

Figure S9. ^{1}H NMR ($CDCl_3$) spectra of compound **1a**.

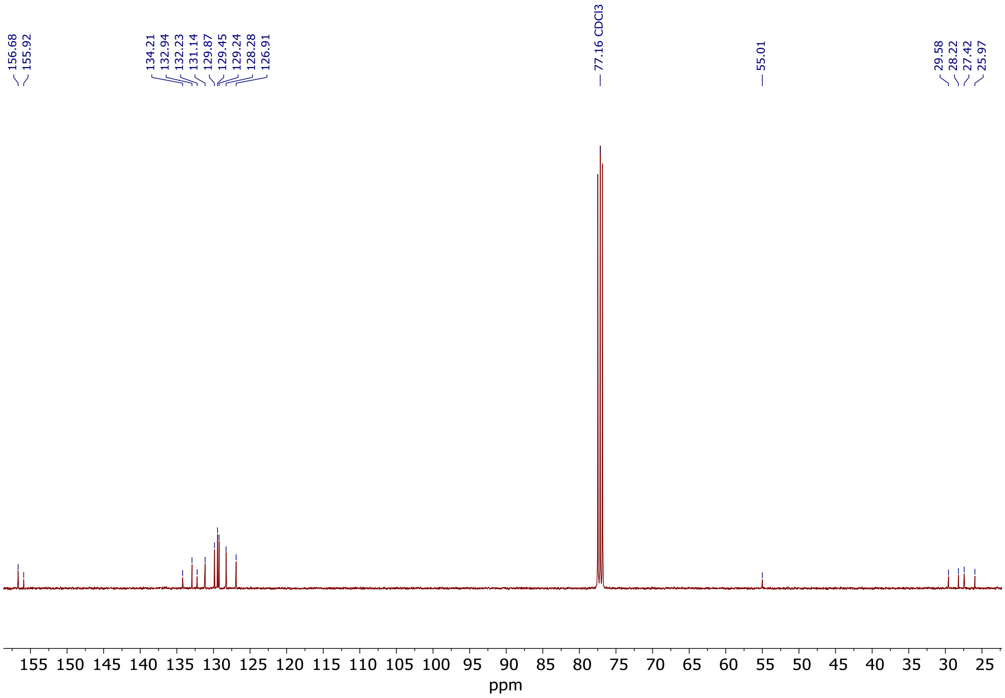

Figure S10. ^{13}C NMR ($CDCl_3$) spectra of compound **1a**.

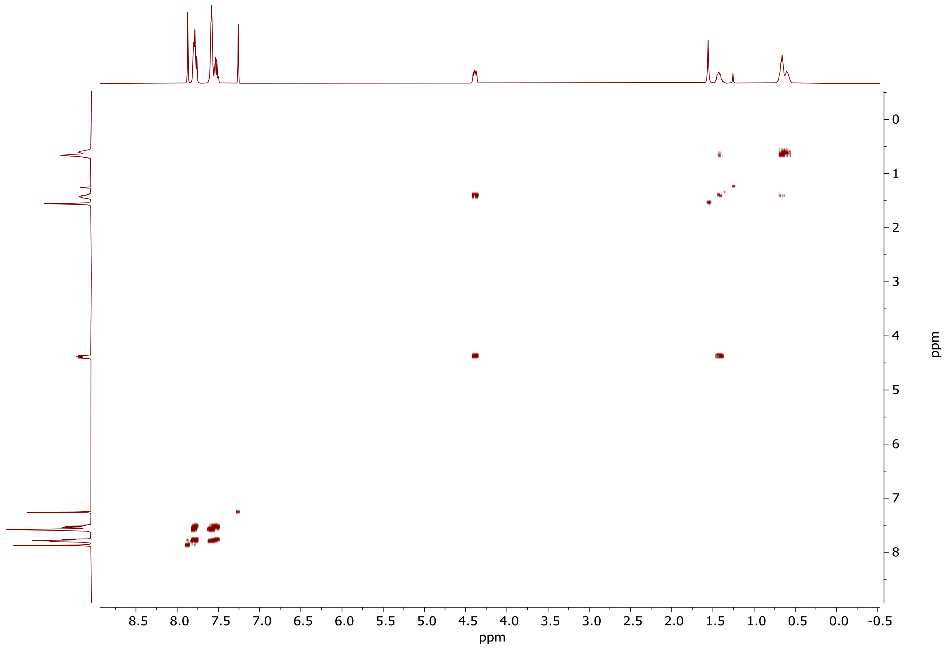

Figure S11. ^{1}H COSY NMR ($CDCl_3$) spectra of compound **1a**.

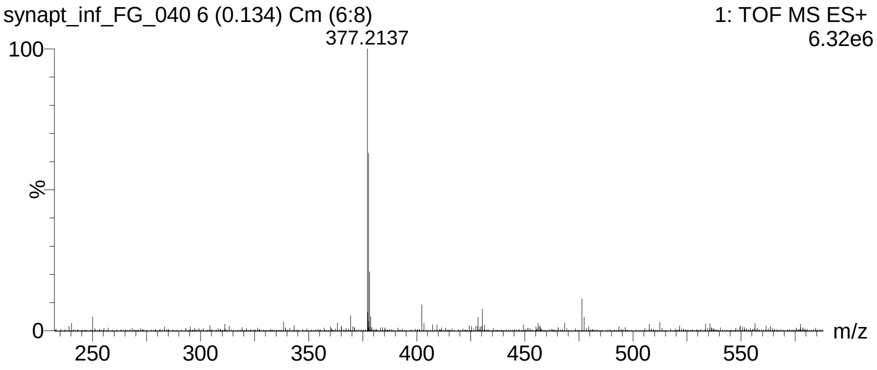

Figure S12. HRMS spectra of compound **1a**.

Compound 1b

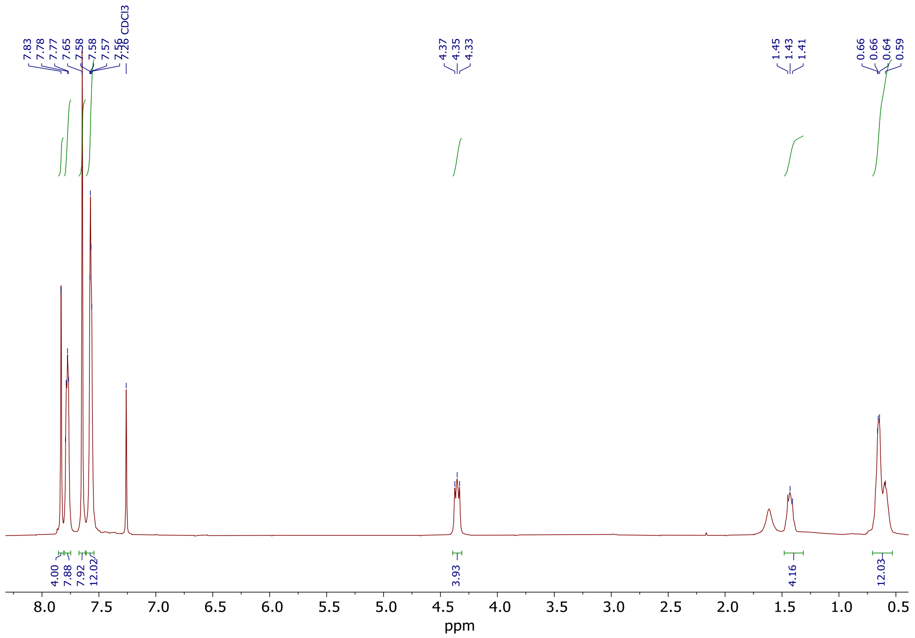

Figure S13. ^{1}H NMR ($CDCl_3$) spectra of compound **1b**.

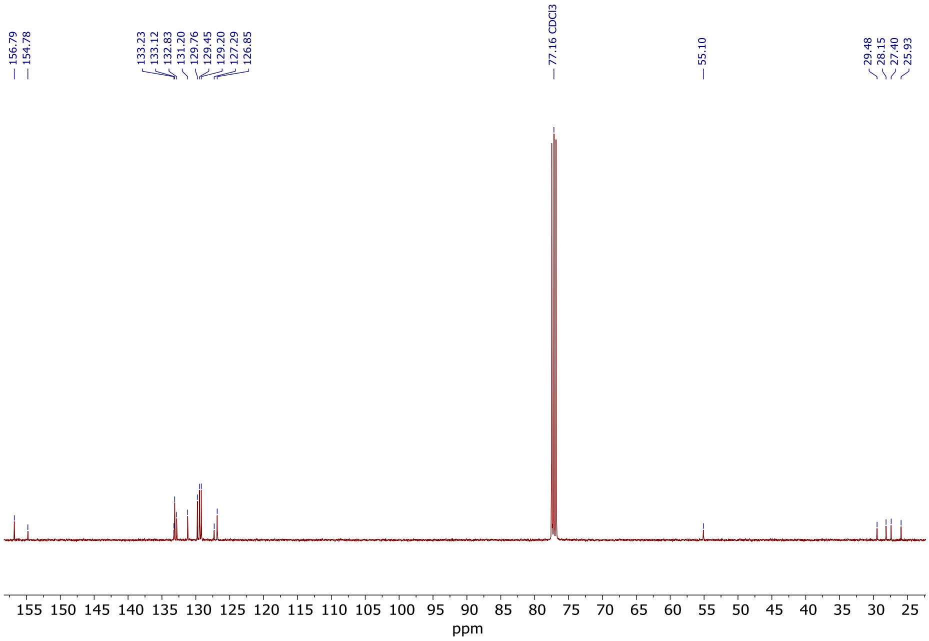

Figure S14. ^{13}C NMR ($CDCl_3$) spectra of compound **1b**.

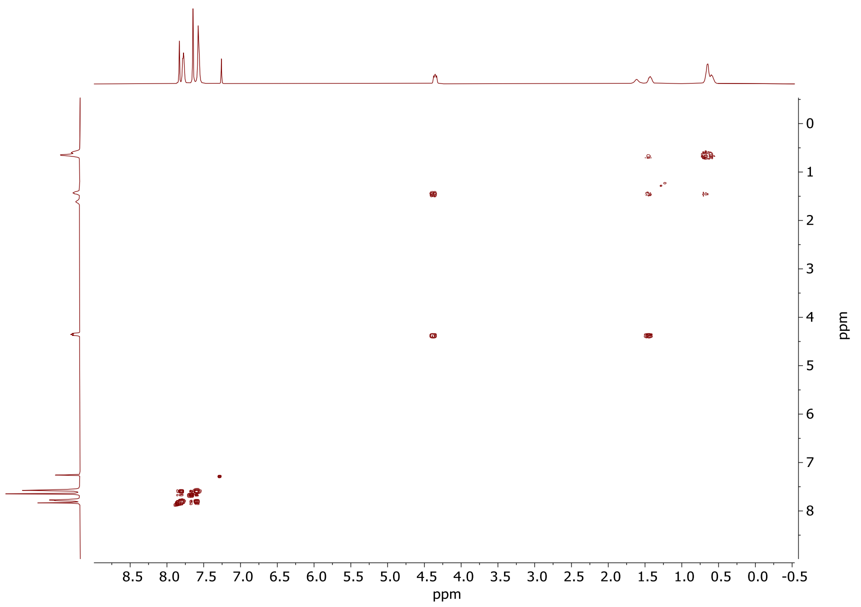

Figure S15. ^{1}H COSY NMR ($CDCl_3$) spectra of compound **1b**.

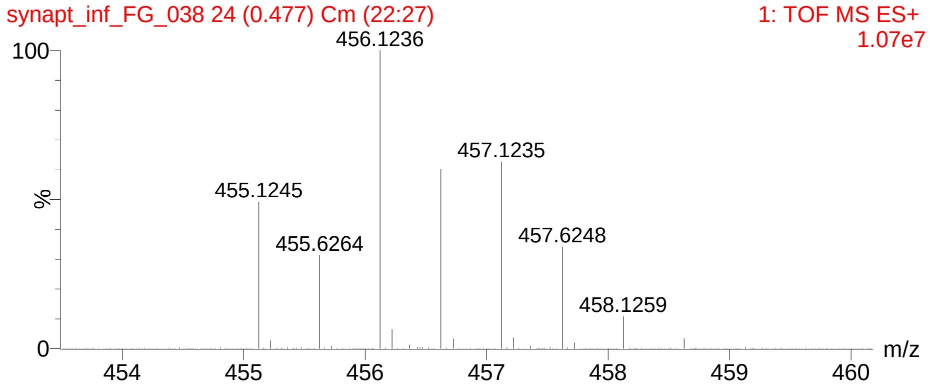

Figure S16. HRMS spectra of compound **1b**.

Compound 1c

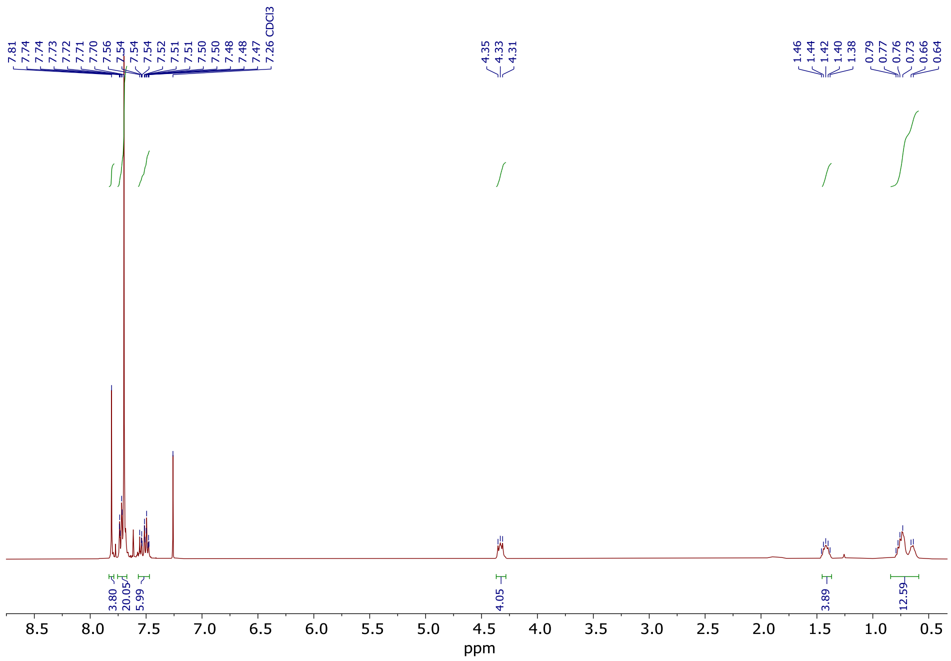

Figure S17. ^{1}H NMR ($CDCl_3$) spectra of compound **1c**.

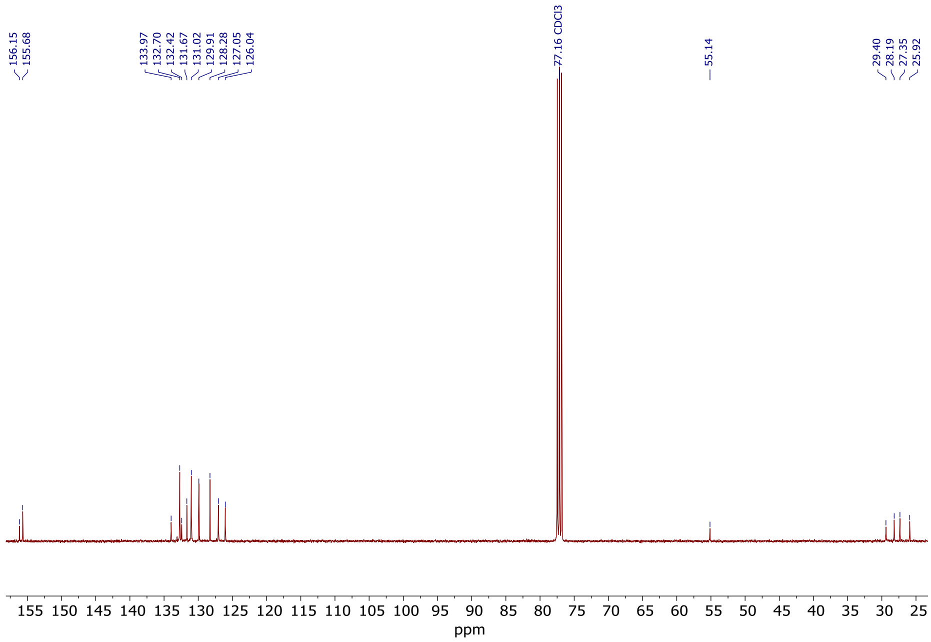

Figure S18. ^{13}C NMR ($CDCl_3$) spectra of compound **1c**.

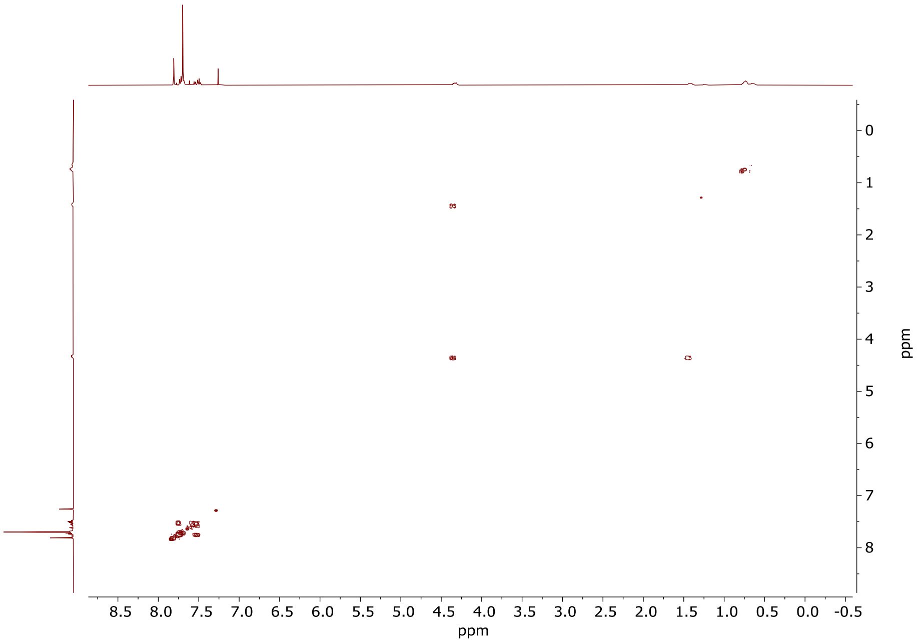

Figure S19. ^{1}H COSY NMR ($CDCl_3$) spectra of compound **1c**.

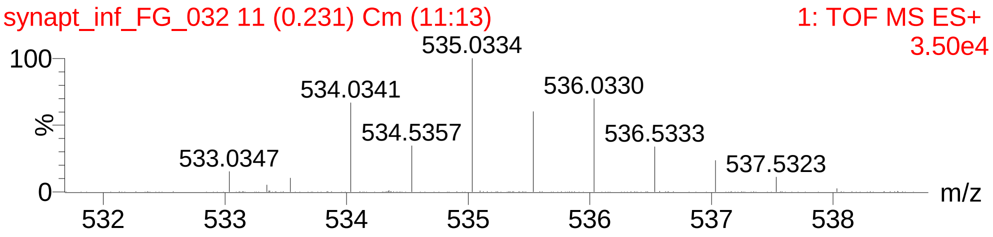

Figure S20. HRMS spectra of compound **1c**.

Compound 1d

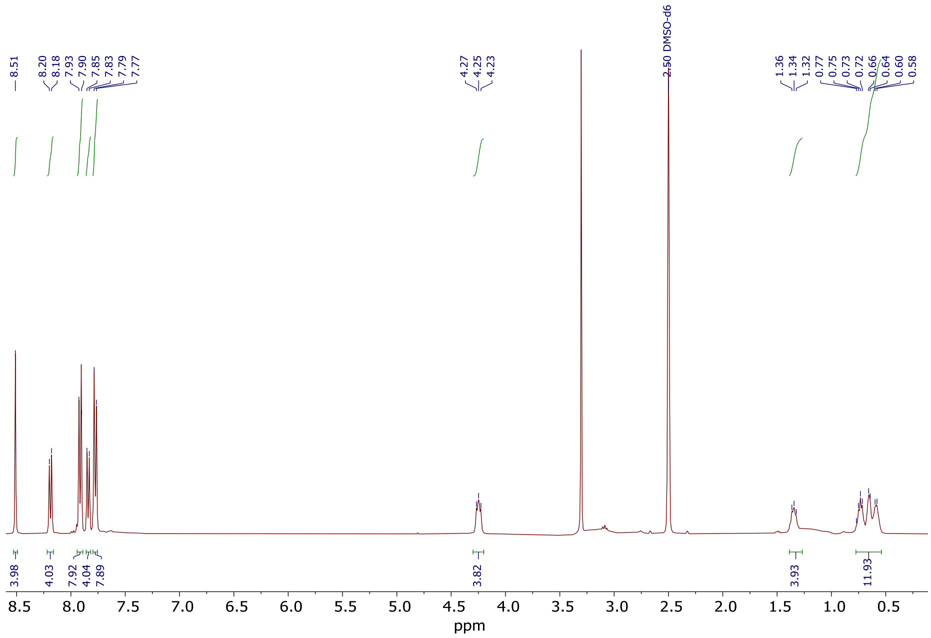

Figure S21. ^{1}H NMR ($CDCl_3$) spectra of compound **1d**.

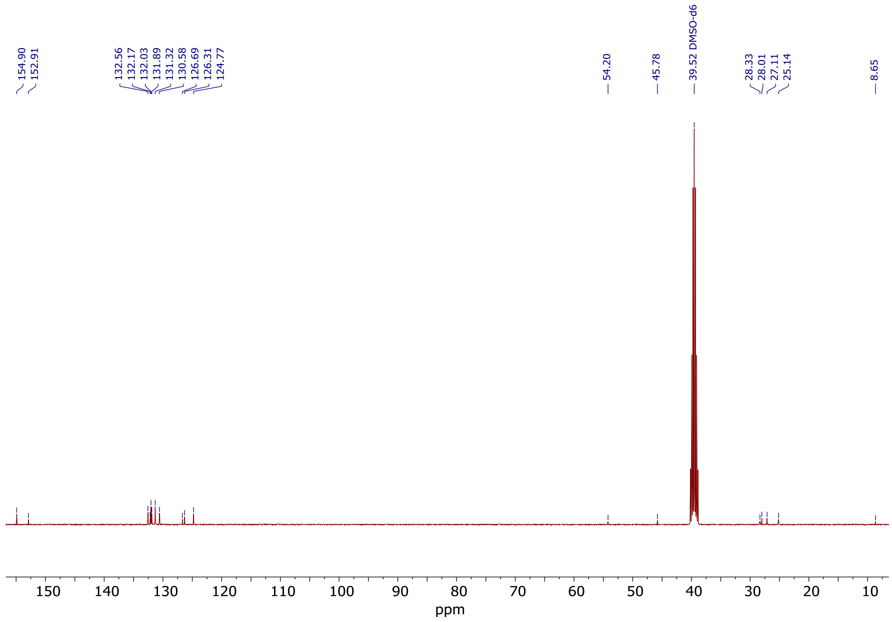

Figure S22. ^{13}C NMR ($CDCl_3$) spectra of compound **1d**.

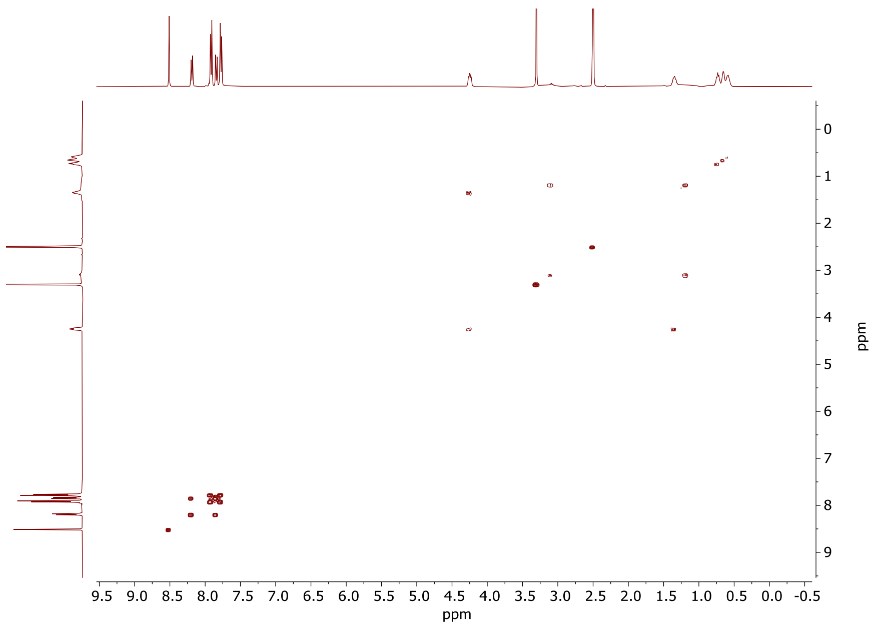

Figure S23. ^{1}H COSY NMR ($CDCl_3$) spectra of compound **1d**.

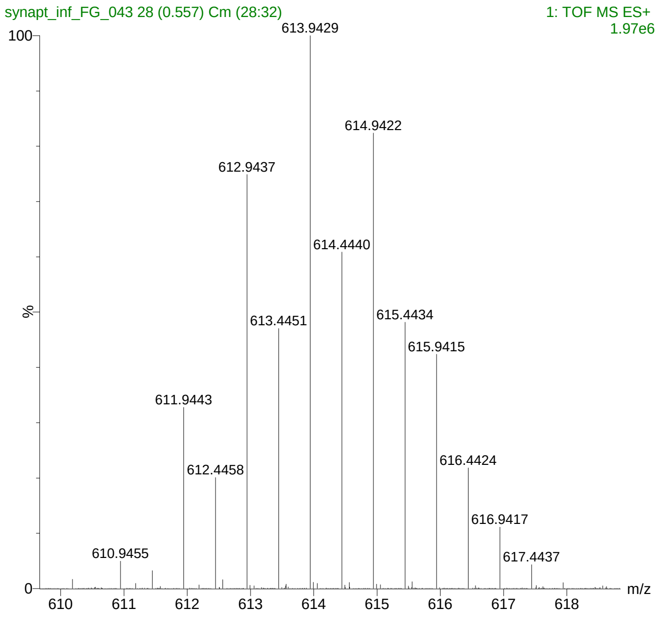

Figure S24. HRMS spectra of compound **1d**.

Compound 2a

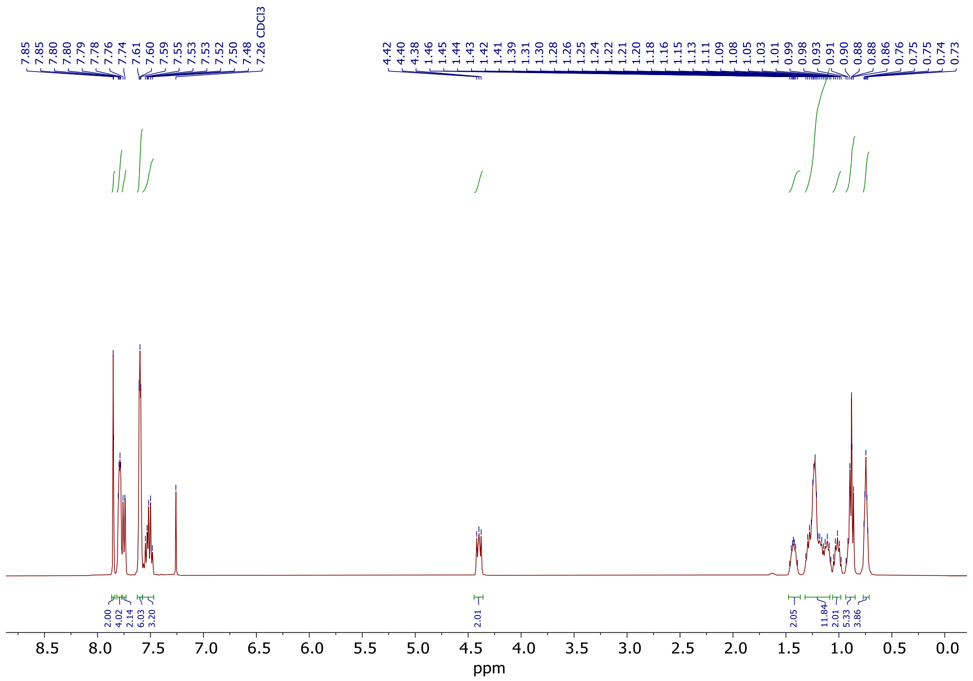

Figure S25. ^{1}H NMR ($CDCl_3$) spectra of compound **2a**.

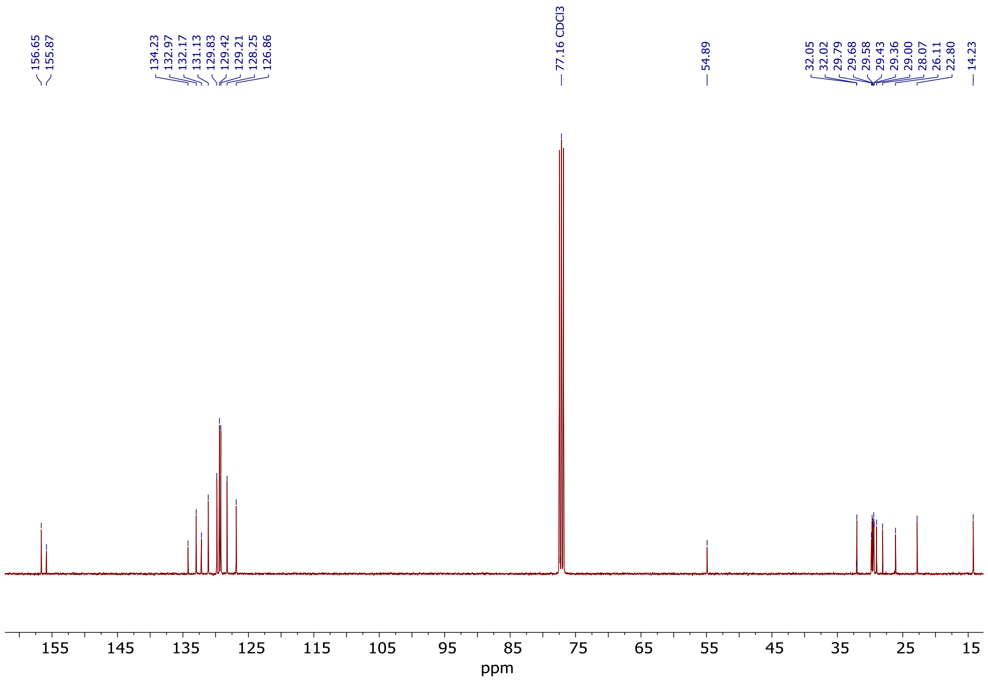

Figure S26. ^{13}C NMR ($CDCl_3$) spectra of compound **2a**.

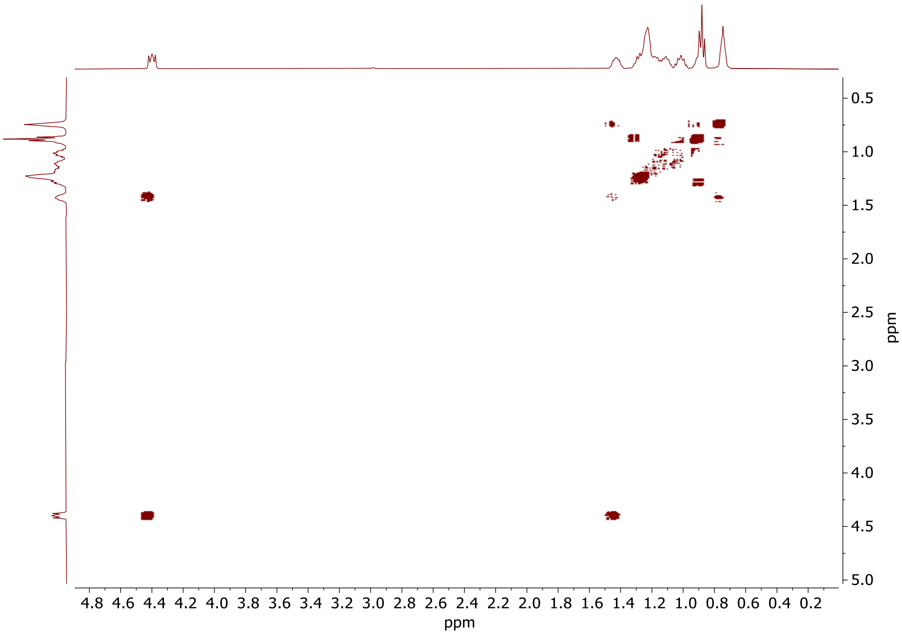

Figure S27. ^{1}H COSY NMR ($CDCl_3$) spectra of compound **2a**.

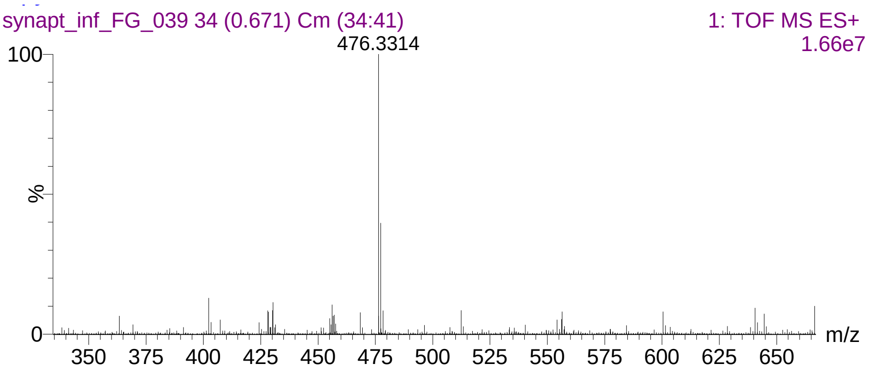

Figure S28. HRMS spectra of compound **2a.**

Compound 2b

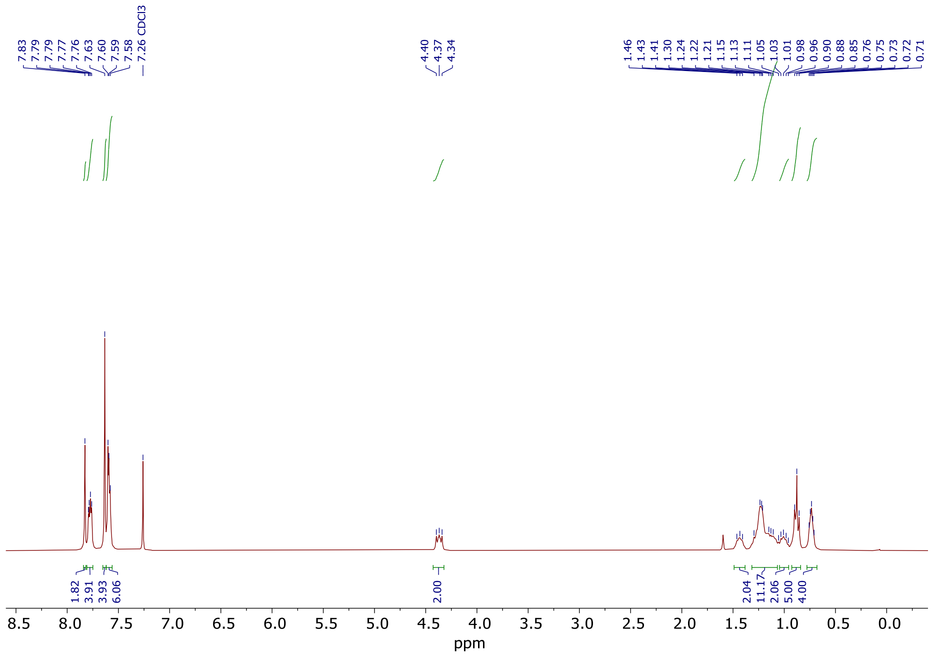

Figure S29. ^{1}H NMR ($CDCl_3$) spectra of compound **2b**.

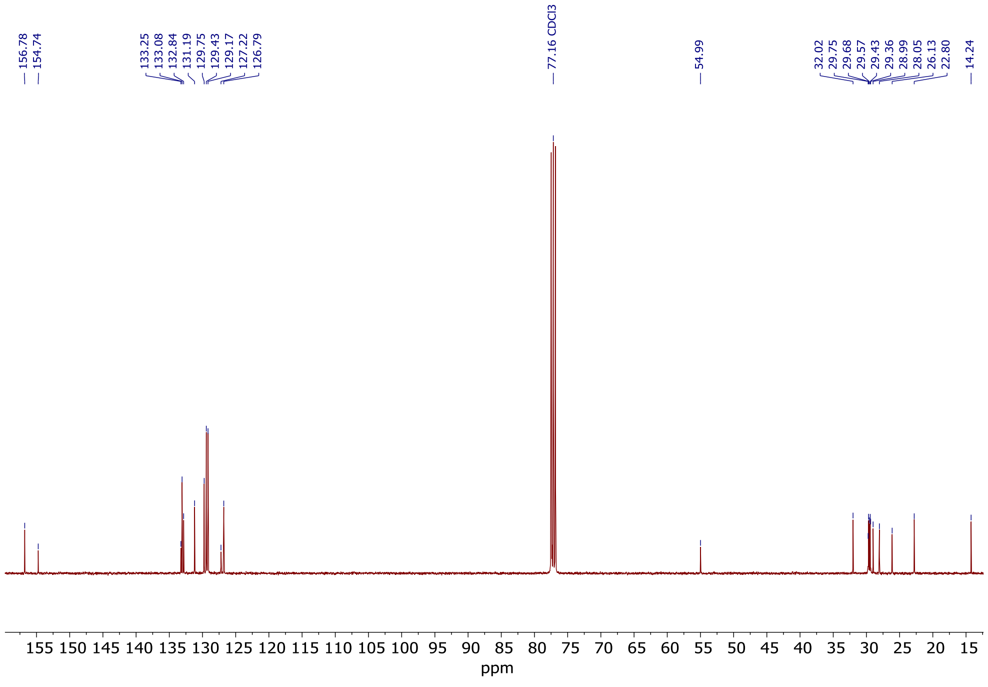

Figure S30. ^{13}C NMR ($CDCl_3$) spectra of compound **2b**.

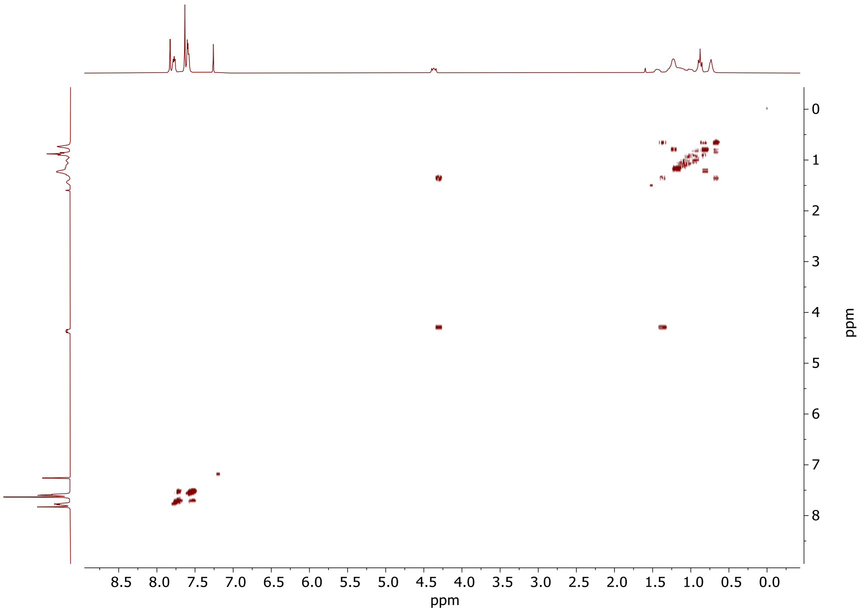

Figure S31. ^{1}H COSY NMR ($CDCl_3$) spectra of compound **2b**.

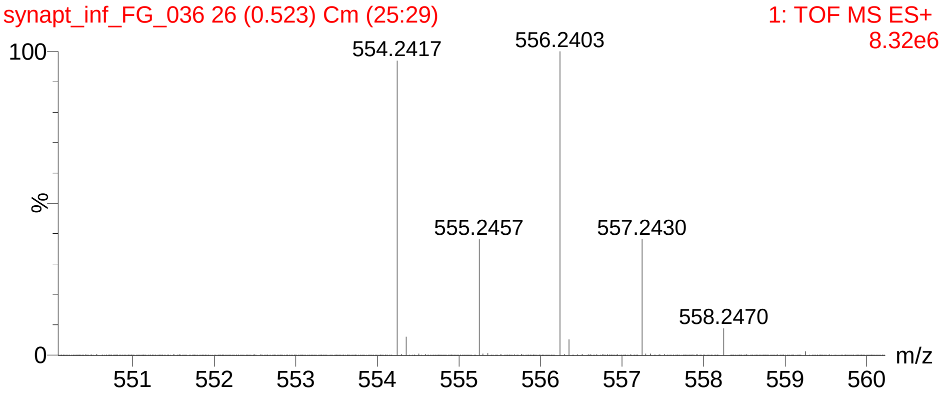

Figure S32. HRMS spectra of compound **2b.**

Compound 2c

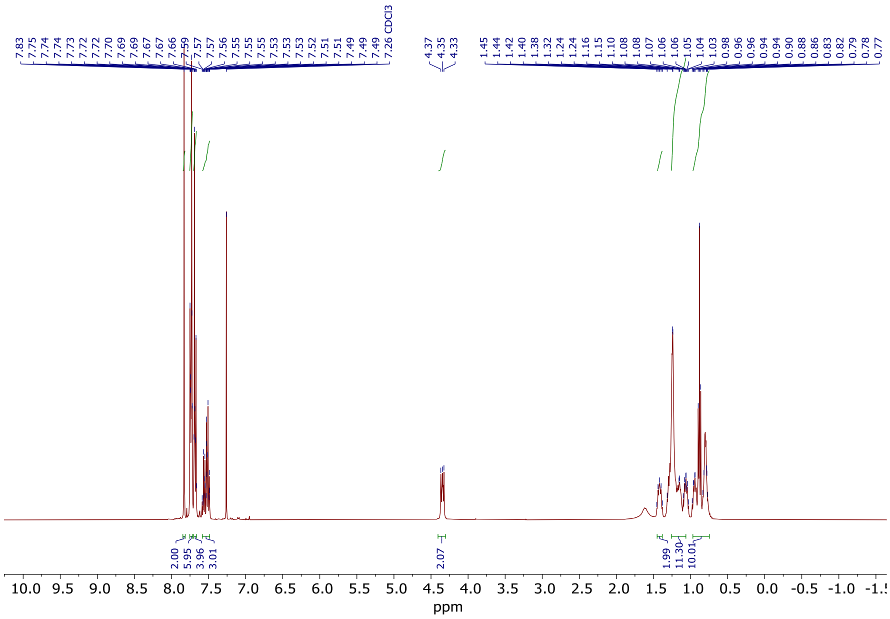

Figure S33. ^{1}H NMR ($CDCl_3$) spectra of compound **2c**.

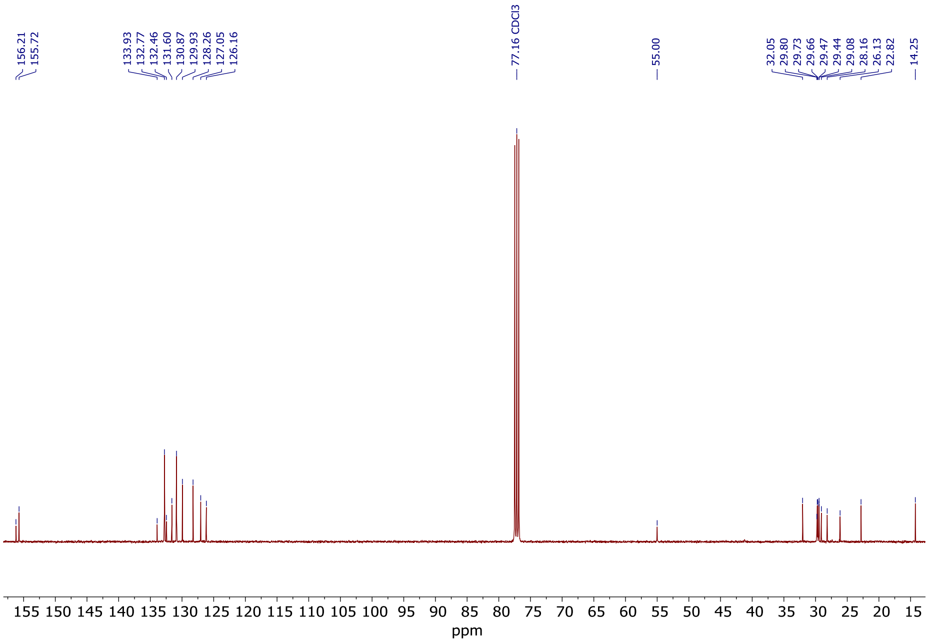

Figure S34. ^{13}C NMR ($CDCl_3$) spectra of compound **2c**.

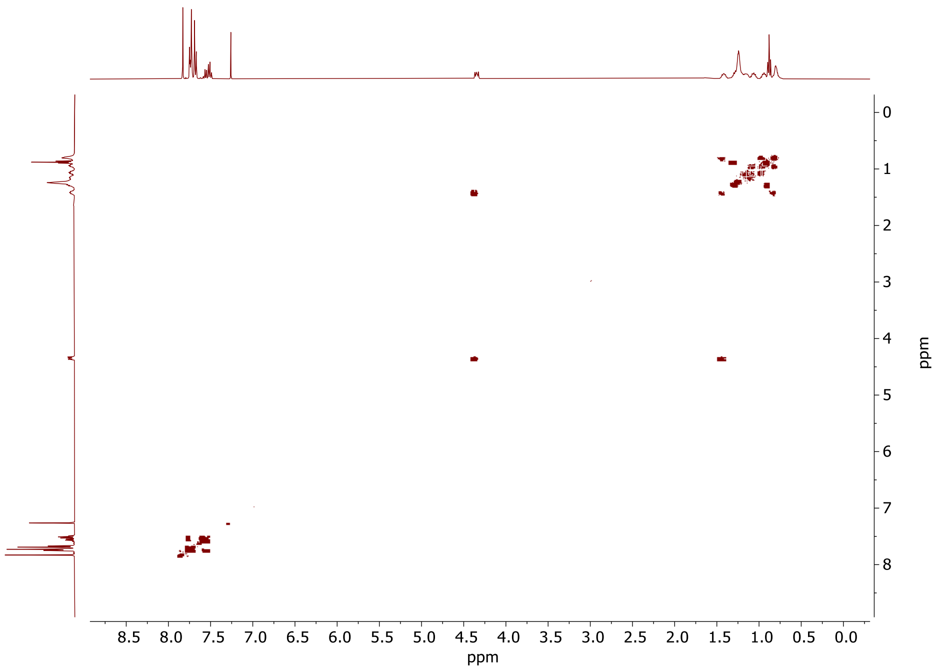

Figure S35. ^{1}H COSY NMR ($CDCl_3$) spectra of compound **2c**.

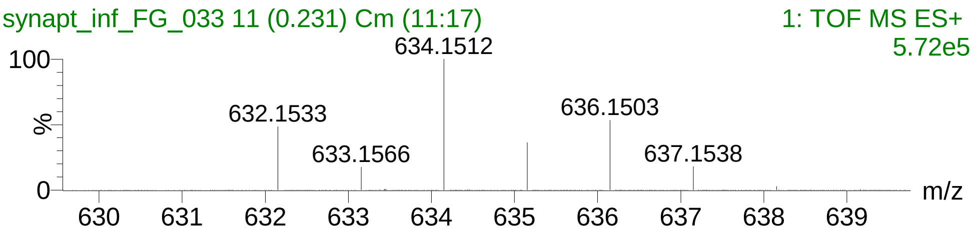

Figure S36. HRMS spectra of compound **2c.**

Compound 2d

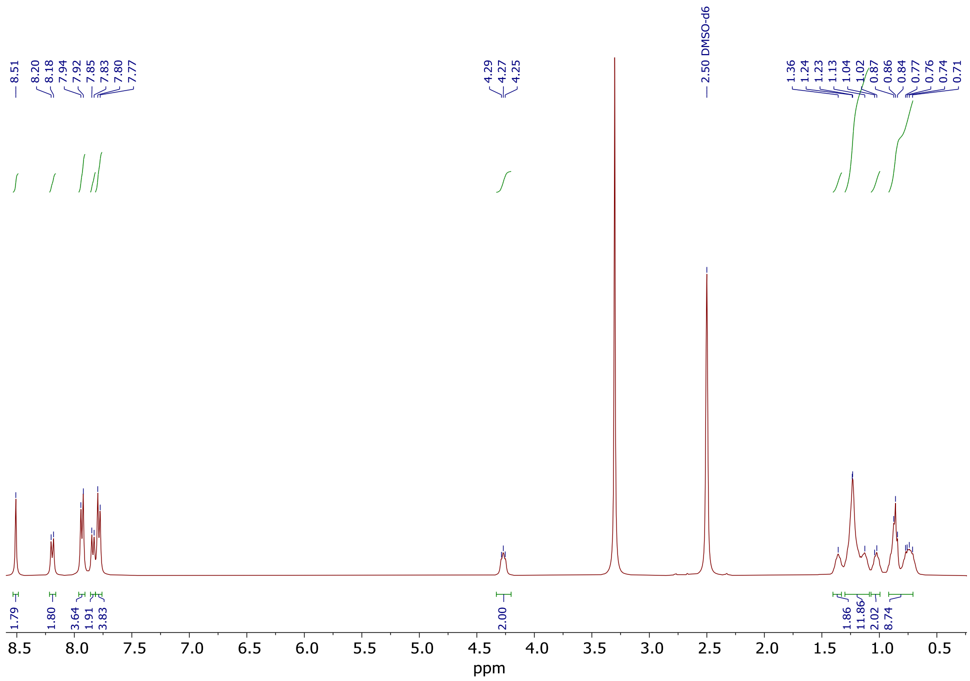

Figure S37. ^{1}H NMR (DMSO-d_6) spectra of compound **2d**.

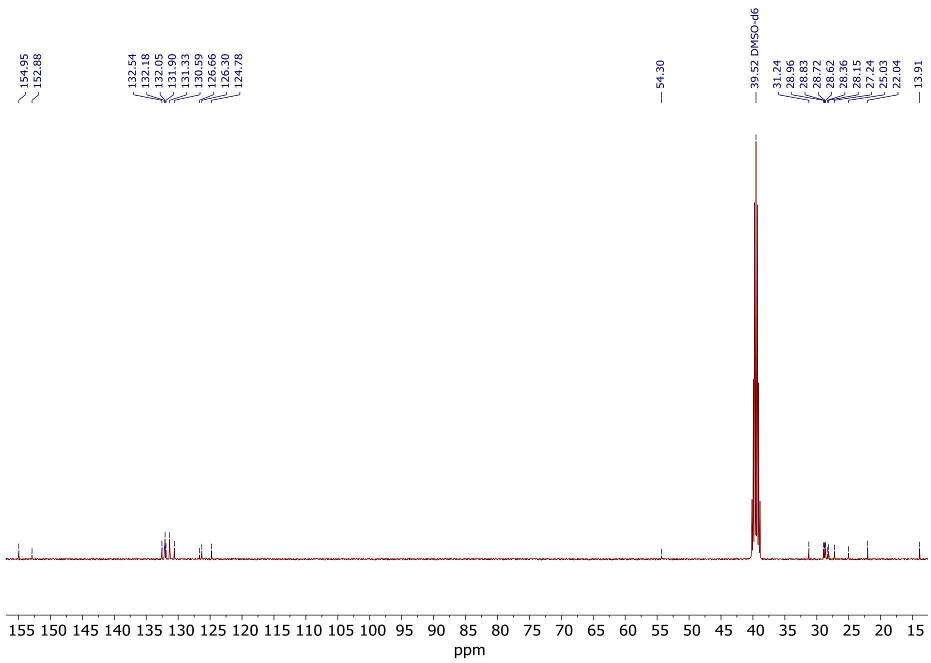

Figure S38. ^{13}C NMR (DMSO-d_6) spectra of compound **2d**.

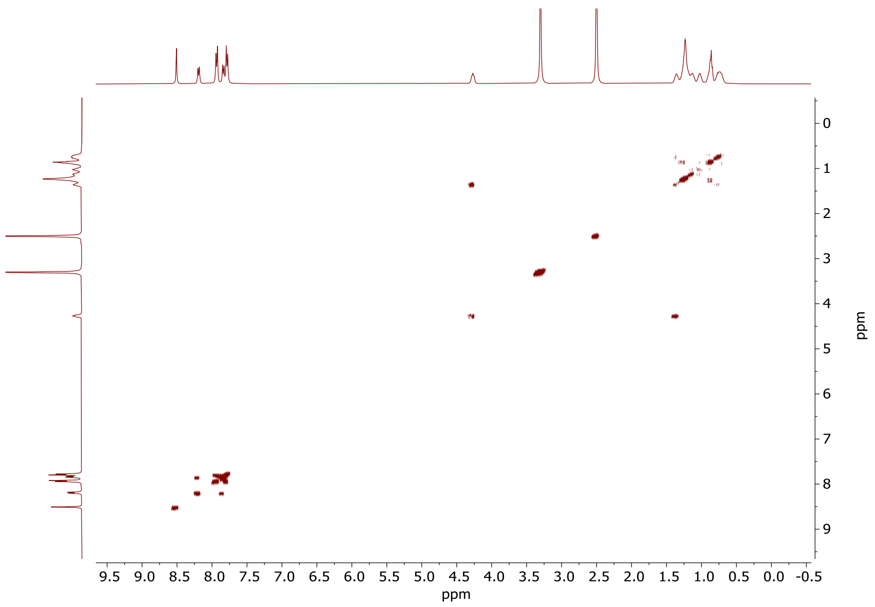

Figure S39. ^{1}H COSY NMR (DMSO-d_6) spectra of compound **2d**.

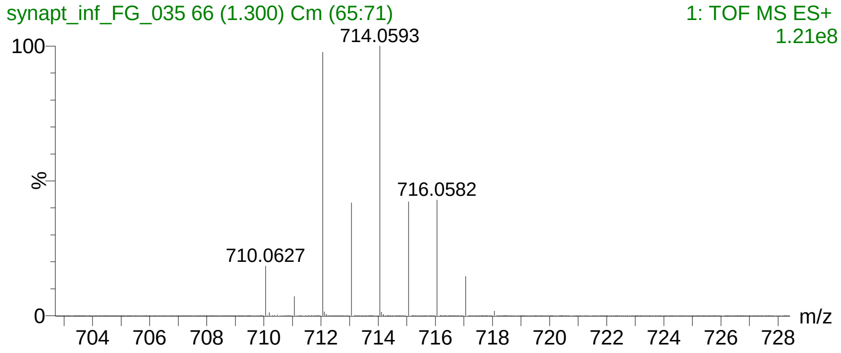

Figure S40. HRMS spectra of compound **2d.**

Compound 3a

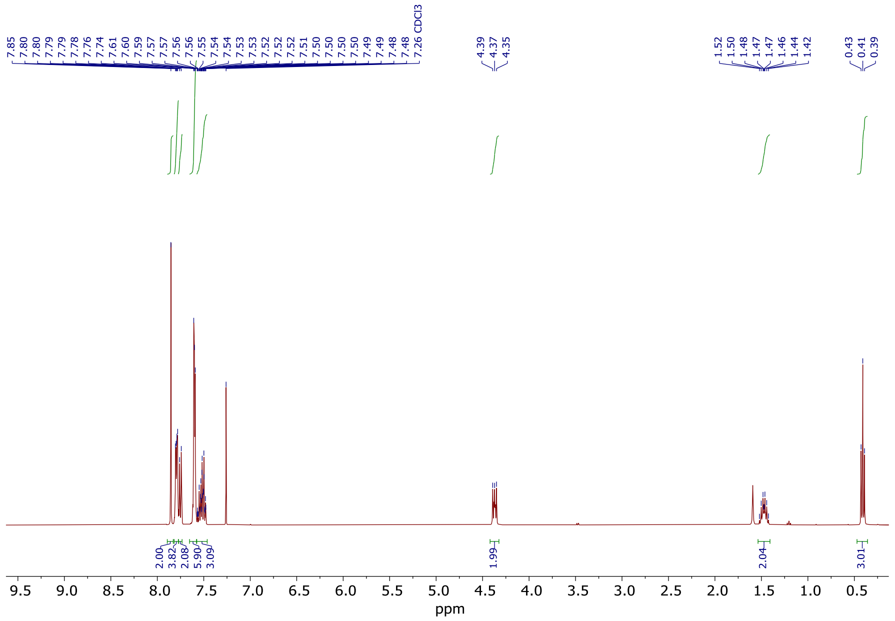

Figure S41. ^{1}H NMR ($CDCl_3$) spectra of compound **3a**.

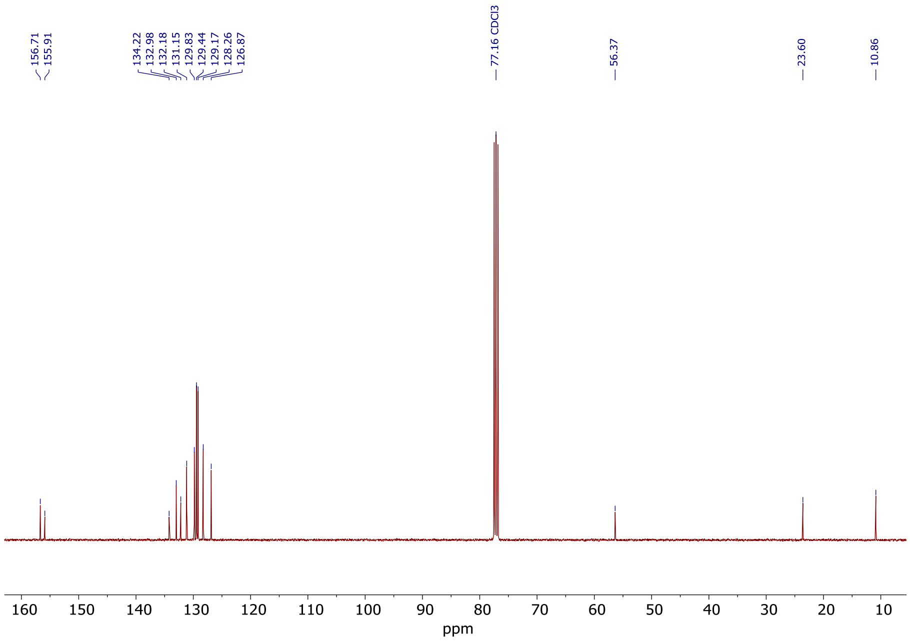

Figure S42. ^{13}C NMR ($CDCl_3$) spectra of compound **3a**.

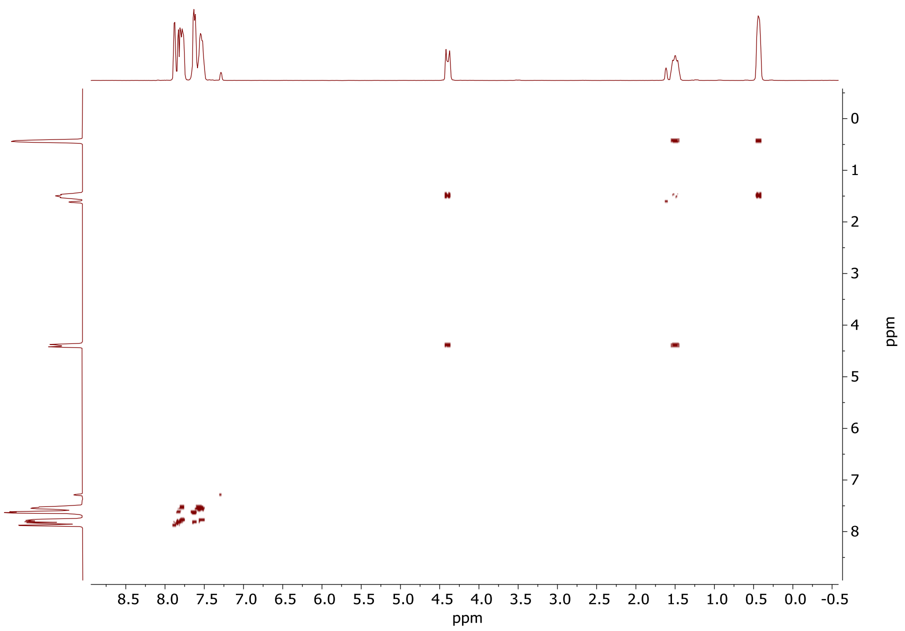

Figure S43. ^{1}H COSY NMR ($CDCl_3$) spectra of compound **3a**.

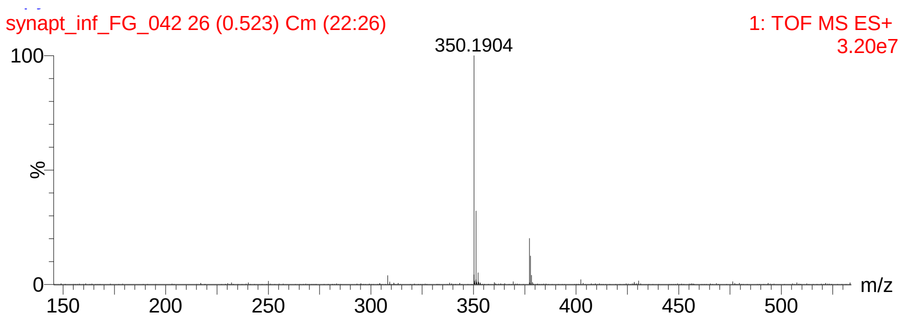

Figure S44. HRMS spectra of compound **3a**.

Compound 3b

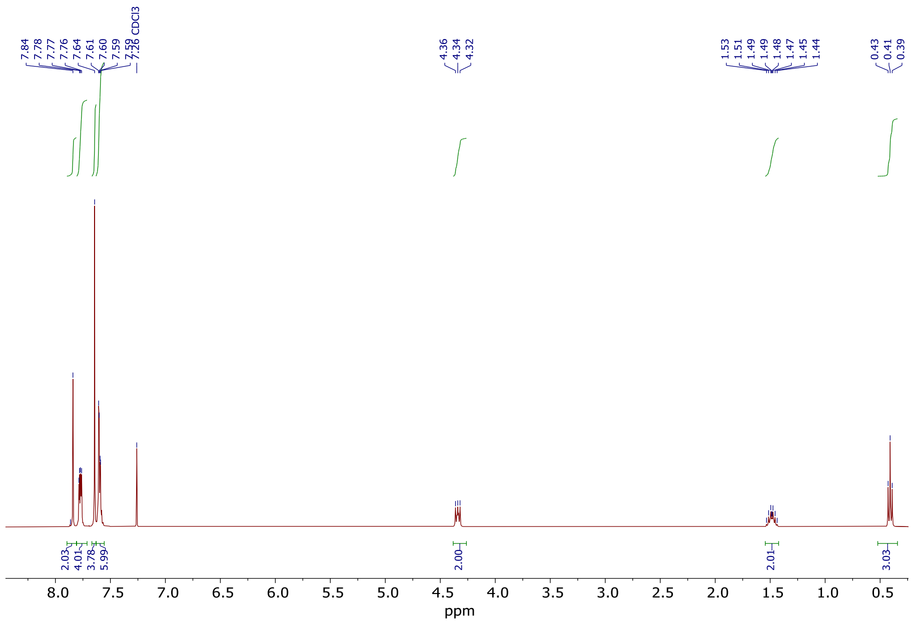

Figure S45. ^{1}H NMR ($CDCl_3$) spectra of compound **3b**.

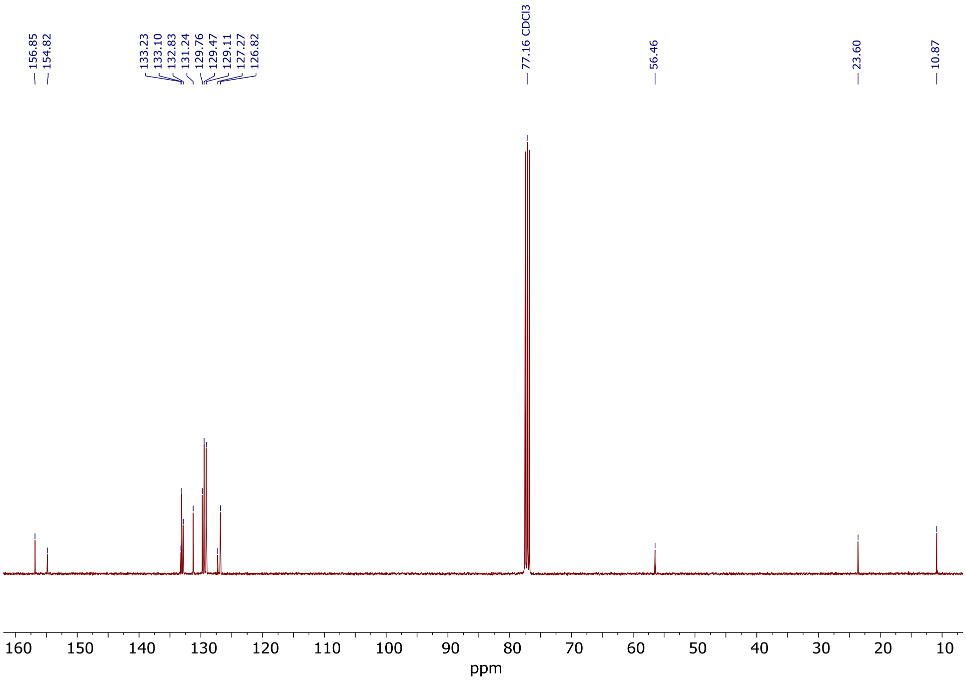

Figure S46. ^{13}C NMR ($CDCl_3$) spectra of compound **3b**.

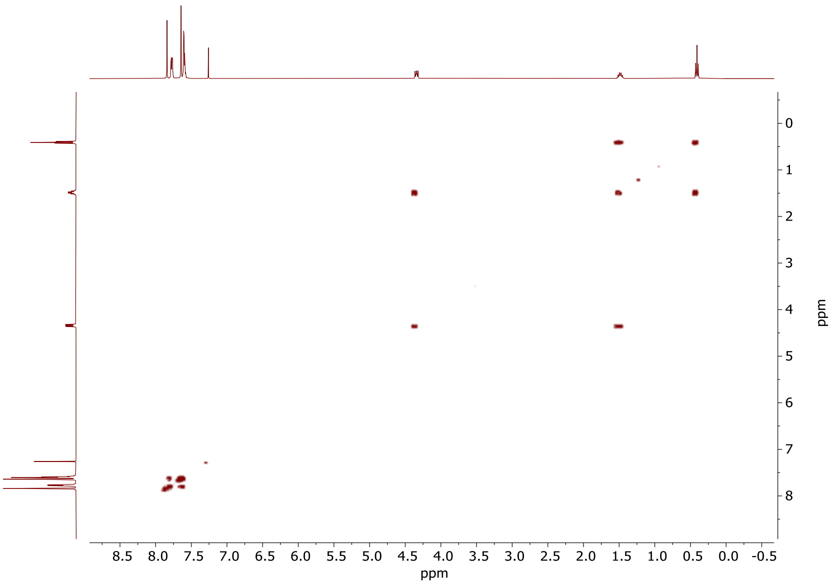

Figure S47. ^{1}H COSY NMR ($CDCl_3$) spectra of compound **3b**.

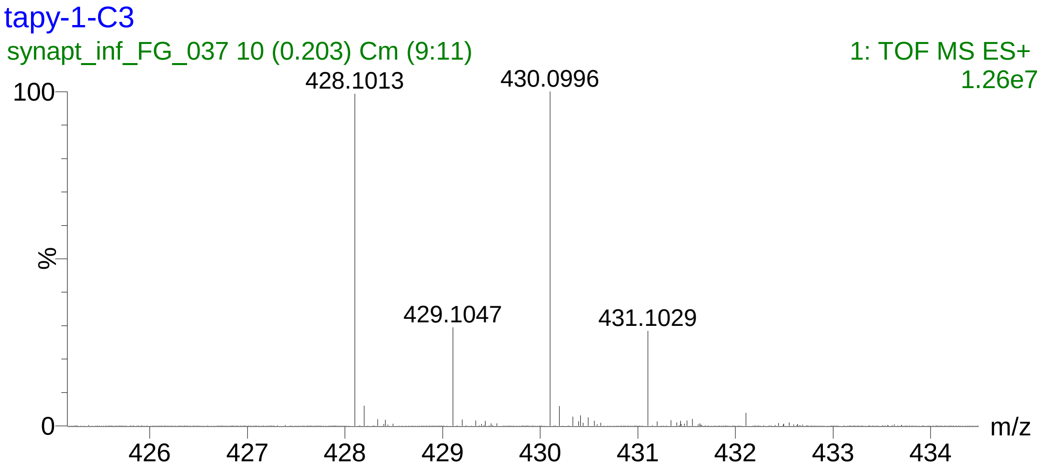

Figure S48. HRMS spectra of compound **3b**.

Compound 3c

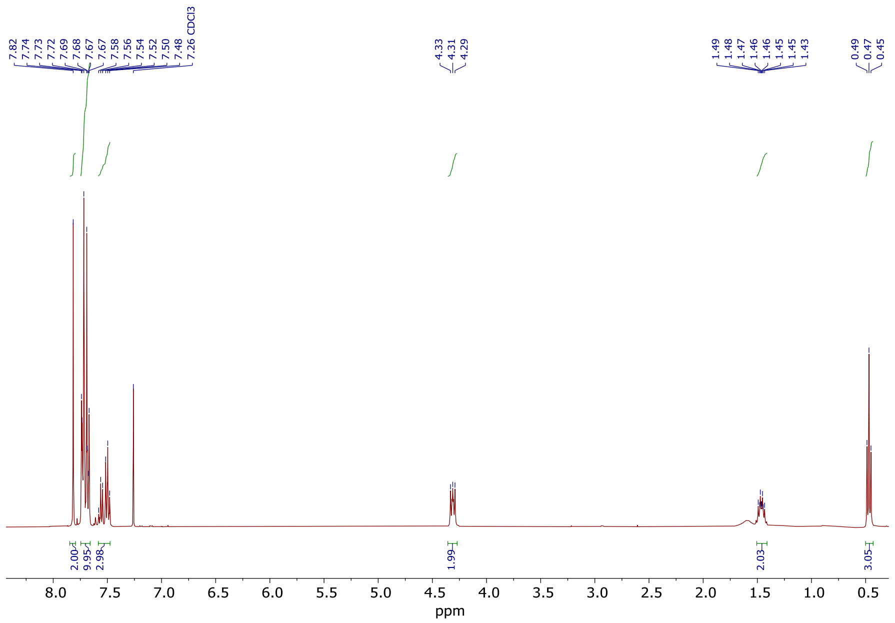

Figure S49. ^{1}H NMR ($CDCl_3$) spectra of compound **3c**.

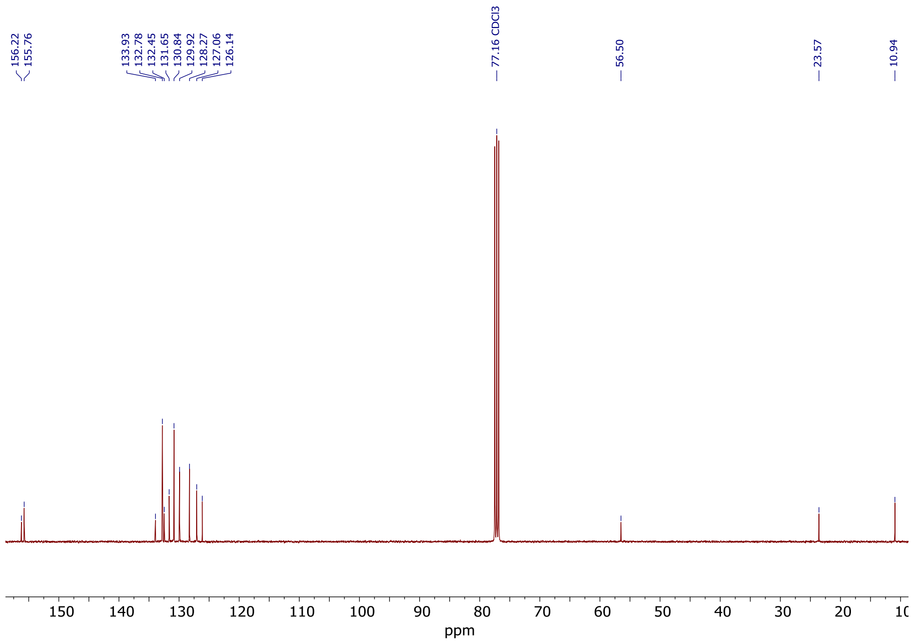

Figure S50. ^{13}C NMR ($CDCl_3$) spectra of compound **3c**.

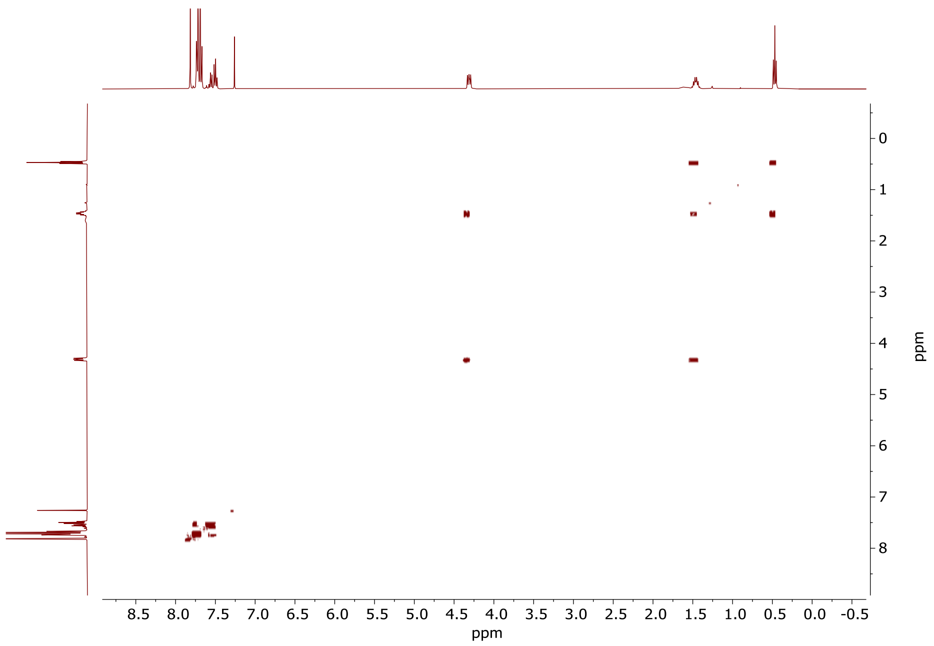

Figure S51. ^{1}H COSY NMR ($CDCl_3$) spectra of compound **3c**.

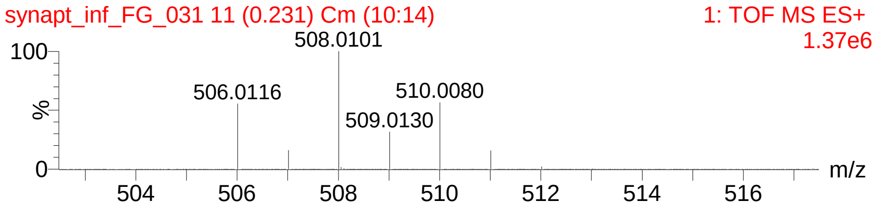

Figure S52. HRMS spectra of compound **3c**.

Compound 3d

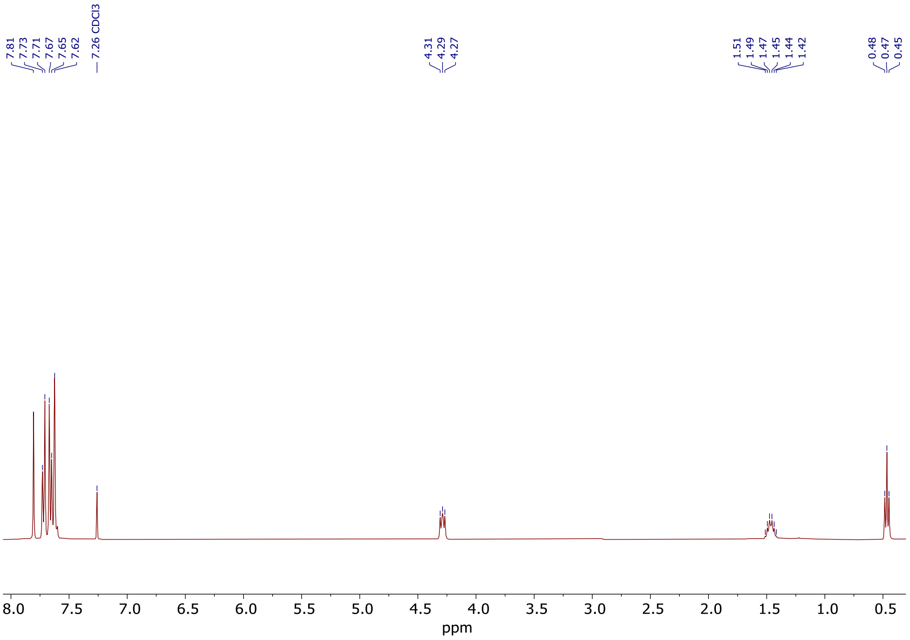

Figure S53. ^{1}H NMR ($CDCl_3$) spectra of compound **3d**.

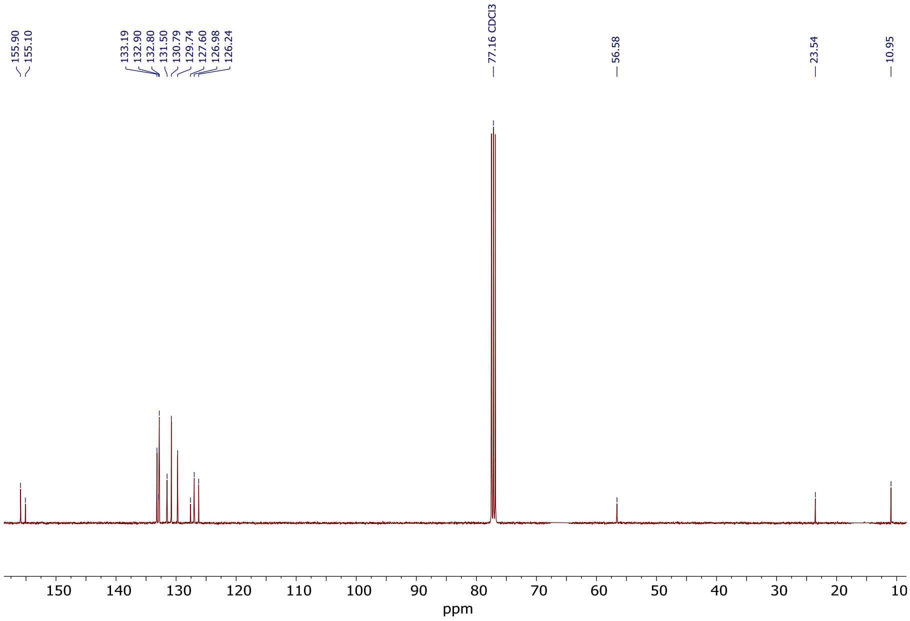

Figure S54. ^{13}C NMR ($CDCl_3$) spectra of compound **3d**.

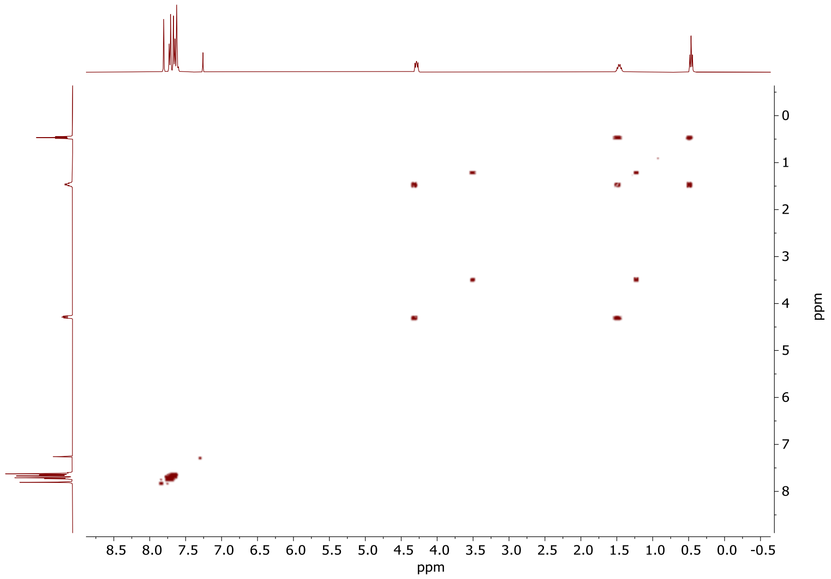

Figure S55. ^{1}H COSY NMR ($CDCl_3$) spectra of compound **3d**.

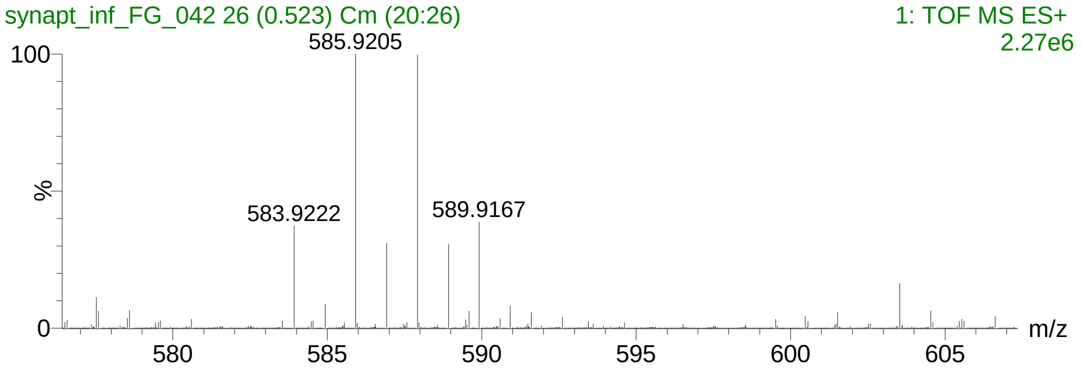

Figure S56. HRMS spectra of compound **3d**.

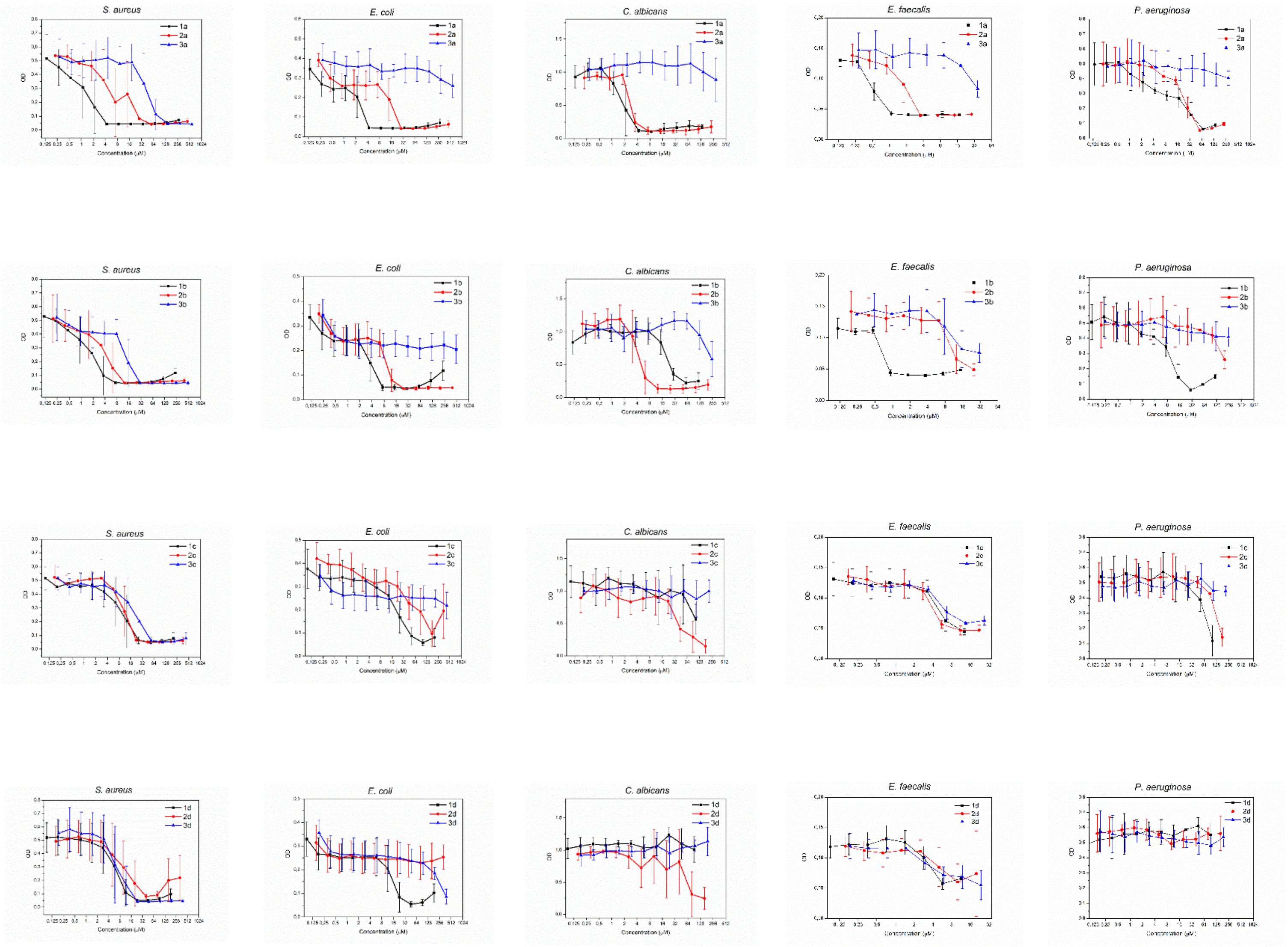

Figure S57. Determination of MICs for all the studied compounds.
